# Supplementary material for: Non-additive microbial community responses to environmental complexity
Source: Nat Commun. 2021 Apr 22;12:2365. doi: 10.1038/s41467-021-22426-3 (PMC8062479; doi:10.1038/s41467-021-22426-3)
Supplement: Supplementary file 1 — Supplementary Information [file 41467_2021_22426_MOESM1_ESM.pdf]

Supplementary Information

Non-additive microbial community responses to environmental complexity

Alan R. Pacheco, Melisa L. Osborne, and Daniel Segrè

Supplementary Note 1

Our observation that the yields of individual organisms and small communities increased with environmental complexity – but those of our larger community remained constant – suggests an interplay between different mechanisms that either enhance or dampen E_Y based on initial community size. On the one hand, community experiments (Supplementary Figure 22) and previous studies[1,2] have indicated that microbial growth efficiency can scale nonlinearly with concentration, and that community growth rates can increase with environmental complexity (Supplementary Figure 23a)[3,4]. Such metabolic nonlinearities may be more dominant in smaller communities given that they can be more commonly dominated by a single organism (Supplementary Figure 20). On the other hand, ecological phenomena such as cross-feeding could make a wider pool of resources available over time, enriching even simple environments[5] and thereby reducing the positive impact that initially complex environments have on yields. Given that they contain a greater variety of organisms, it may be that larger communities allow for the accumulation of more of these resources, potentially explaining the lack of skewness in E_Y. This notion is supported by our observation that the yields of a different 13-species community grown in fewer carbon sources (com13a) did not significantly increase with environmental complexity (Supplementary Figure 23b), as well as by the distribution of E_Y for com13 being skewed at earlier experimental timepoints (Supplementary Figure 24). In addition, we observed evidence of possible byproduct utilization in the form of diauxic shifts in batch culture experiments (Supplementary Figure 3a, Supplementary Figure 22a-c). Nonetheless, it is not clear whether the number of secreted metabolic byproducts would be expected to increase significantly with environmental complexity. In fact, stoichiometric modeling (Supplementary Methods) suggested that the number of secreted metabolites quickly plateaus as the number of resources increases Supplementary Figure 25). Furthermore, such an amplification of the space of available resources would result in increased taxonomic diversity[1,6], which was not observed experimentally (Figure 3a, Supplementary Figure 20).

Supplementary Note 2

Given the taxonomic variability observed across our dataset, we suspected that unrelated carbon source combinations could nonintuitively yield similar taxonomic compositions. A hierarchical clustering of all 63 carbon source combinations revealed such environment-phenotype pairings (Supplementary Figure 14), which also resulted in the emergence of three distinct organism groupings (Supplementary Figure 15). These groupings, which resemble previously-identified family-level functional relationships in natural communities[5], provide insight into the types of carbon sources that need to be present to favor a particular taxon. They do not, however, explain how individual carbon sources behave in higher-order combinations. We therefore generated a linear model to determine whether any particular carbon sources were more universally associated with higher taxonomic diversity. Indeed, although we identified a number of such carbon sources, we still found that their effects could be eclipsed by those that disproportionately favored a single organism (Supplementary Table 6).

Supplementary Note 3

In addition to modeling statistical ensembles of communities, we applied our consumer resource model to simulate the taxonomic compositions of com3 and com4, which were strikingly low in biodiversity and often dominated by the organisms with the broadest resource utilization capabilities (Supplementary Figure 20). Our model was parametrized with experimentally-obtained growth data (Supplementary Figure 9a) and featured the potential for cross-feeding of secreted byproducts[5,7] informed by flux-balance predictions of metabolic turnover (Supplementary Methods, Supplementary Figure 25a). This parametrization enabled us to make quantitative

estimates of community growth trajectories and metabolic exchange (Supplementary Figure 26), yielding accurate predictions of the dominance of *P. aeruginosa* – which had the broadest set of usable carbon sources – across most conditions in com4 (Supplementary Figure 27). However, our model could not fully explain the dominance of *S. oneidensis* in com3 or the failure of *M. extorquens* to grow in mixed culture despite having relatively broad resource utilization capabilities in monoculture (Supplementary Figure 9). Despite these inaccuracies, our experimentally-parametrized model recapitulated the low levels of taxonomic diversity we observed *in vitro*.

Supplementary Methods

Consumer resource modeling of experimental communities. In addition to using our consumer resource models (CRMs) for simulating statistical ensembles of communities (see Methods), we applied it to explicitly model our three- and four-species communities (com3 and com4) To do this, we parametrized the resource preference matrix C based on the growth yields of each individual organism on our 32 individual carbon sources (Supplementary Figure 9a). We used flux-balance models for each of the four organisms[8–11] to determine the fraction of each resource α that could be converted to a secreted metabolite β (Supplementary Methods). The secretion fluxes of metabolic byproducts from the organisms under each of the 32 individual resources were calculated using these models, and the ratio of secretion to intake fluxes was used to populate the D matrix. This matrix was then normalized across each primary resource to ensure conservation of mass. Resource uptake efficiencies for these secreted metabolites were then assigned according to the same monoculture growth data used to define preferences for the primary resources. As with our initial arbitrary communities, initial species abundances were set to 6×10^6 CFU/mL and initial resource abundances were set to 1.5 g/mL. We then simulated the growth and potential metabolic exchange of all four community members in coculture over the course of 288 hours with a timestep of 0.01 hours in the 63 environmental conditions for com3 and com4 (Supplementary Table 3).

Flux balance modeling. To estimate the number of secreted and absorbed metabolites in com4, we used experimentally-validated genome-scale models for each of the four organisms[8–11]. Genome-scale models are mathematical representations of the known metabolic capacities of individual organisms[12]. They are constrained by known maximum metabolic fluxes v_{max} through internal and transport reactions, as well as by reaction stoichiometric constraints represented by a matrix S. Flux balance analysis (FBA), a mathematical optimization technique, can then be applied to the models in order to define the metabolic fluxes v within the organism's network that will maximize a particular objective, such as growth[13]. This technique allows us to interrogate the growth rate of organisms under specific environmental conditions, as well as rates of carbon source consumption and metabolite secretion.

Our application of FBA uses the COBRA toolbox[14] and is largely based off of an implementation used in a previous study[1]. Here, we employed FBA to simulate the growth of the four organisms in com4 in the 63 combinatorial medium conditions we tested experimentally. We first defined an *in silico* M9 minimal medium consisting of the various inorganic molecules present in the *in vitro* minimal medium (Supplementary Table 8). These molecules were provided to the genome-scale models at nonlimiting availabilities by setting the corresponding maximum flux bounds v_{max} to 1000 mmol/gDW/hr. Depending on the environmental condition, we supplied each *in silico* organism with the appropriate carbon sources by setting the corresponding maximum flux bounds v_{max} to 10 mmol/gDW/hr. We then applied FBA by maximizing the growth rate and minimizing the sum of the fluxes in the network. This latter step was employed in order to more closely model proteome usage and minimize metabolite cycling throughout the network[15]. The optimization problem applied is therefore:

$$\min|v|,$$
$$\text{s.t.:}$$
$$S \cdot v = 0,$$
$$v_{min} \leq v \leq v_{max}.$$

If an *in silico* organism grew on a given environmental condition, we recorded which organic metabolites were predicted to be taken up and secreted. These are summarized for all environments in Supplementary Table 9.

Supplementary Figures

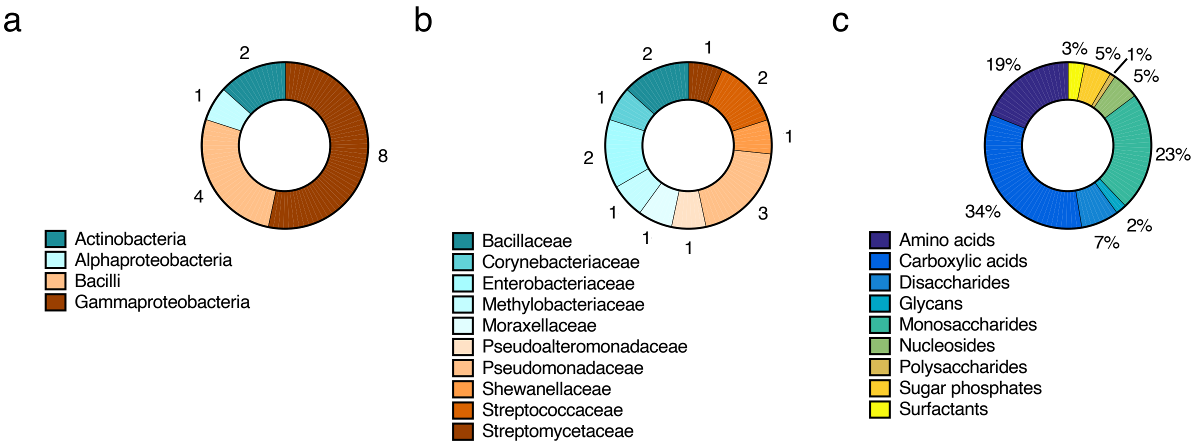

Supplementary Figure 1. Higher-level groupings of organisms and carbon sources used in experiments. **(a-b).** Phylum- **(a)** and family-level **(b)** groupings of 15 bacterial organisms. **(c).** Categories for 95 carbon sources.

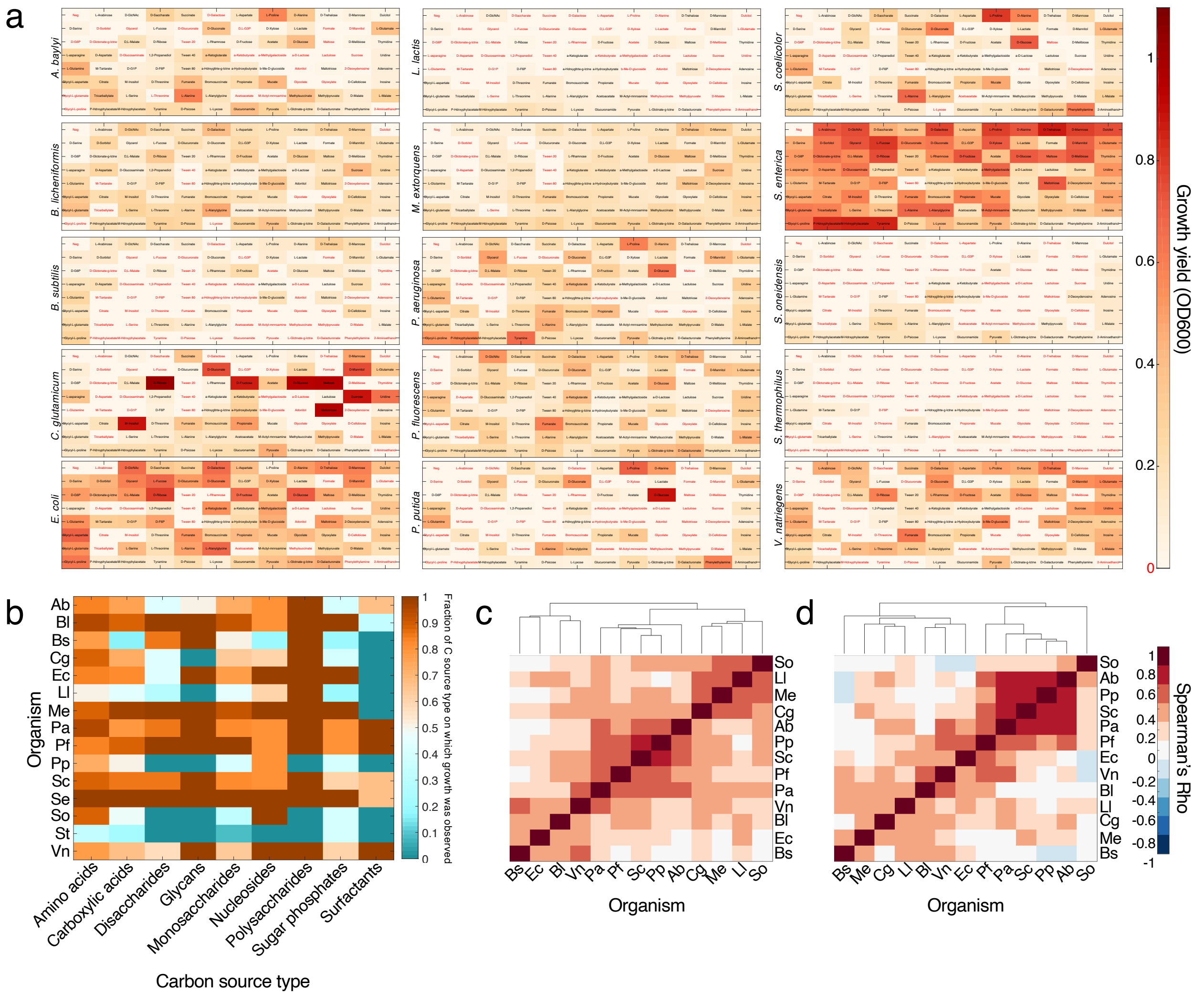

Supplementary Figure 2. Results of Biolog phenotypic assay. **(a).** Mean (3-biological replicate) species-specific growth yields on Biolog carbon sources in M9 minimal medium after 48 hours. Raw OD600 values were corrected for liquid evaporation and a significance cutoff was applied to determine growth above the levels of the negative controls (see Methods). Carbon sources on which growth was not observed are marked in red. **(b).** Growth capabilities of all 15 organisms on 95 Biolog carbon sources. Values displayed are the fraction of a specific carbon source type on which an organism displayed growth. **(c, d).** Hierarchical clustering of 13 selected organisms based on Spearman correlations of growth profiles on all 95 **(c)** and on 32 selected carbon sources **(d)**. Organisms are abbreviated as: Ab: *A. baylyi*, Bl: *B. licheniformis*, Bs: *B. subtilis*, Cg: *C. glutamicum*, Ec: *E. coli*, Ll: *L. lactis*, Me: *M. extorquens*, Pa: *P. aeruginosa*, Pf: *P. fluorescens*, Pp: *P. putida*, Sc: *S. coelicolor*, So: *S. oneidensis*, Vn: *V. natriegens*.

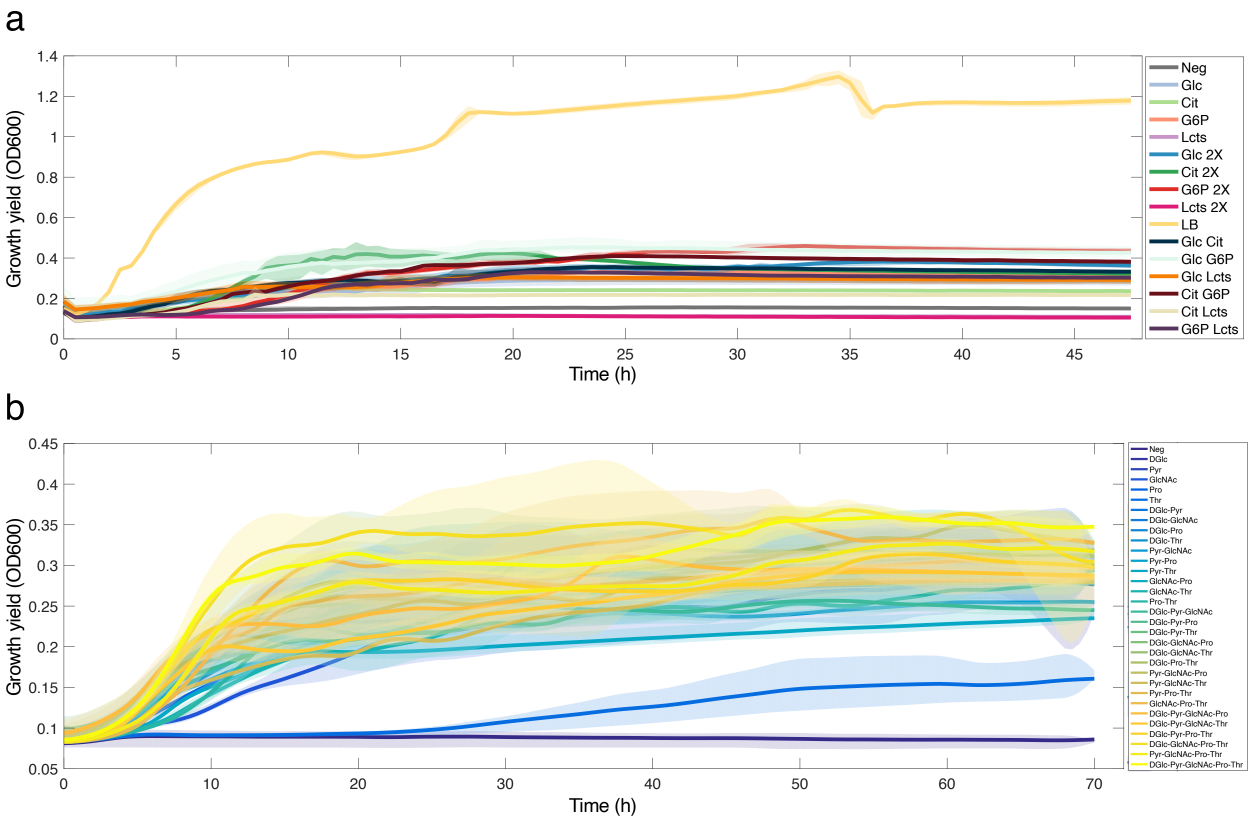

Supplementary Figure 3. Growth curves for multispecies communities in combinatorial environments. **(a).** Growth trajectories of 14-species communities (com14, Supplementary Table 5) on combinations and different concentrations (25 mM C and 50 mM C) of D-glucose (Glc), citrate (Cit), glucose-6-phosphate (G6P), and a-D-lactose (Lcts). Double-carbon source conditions contain 50 mM C of total carbon source, for 25 mM C of each individual carbon source. **(b).** Growth trajectories of 13-species community (com13a) on equimolar concentrations (50 mM C) of five carbon sources: D-glucose (DGlc), pyruvate (Pyr), GlcNAc (GlcNAc), L-proline (Pro), and L-threonine (Thr). Lines in both plots indicate the mean growth yield, and shaded regions in both plots indicate standard deviations across three biological replicates per condition.

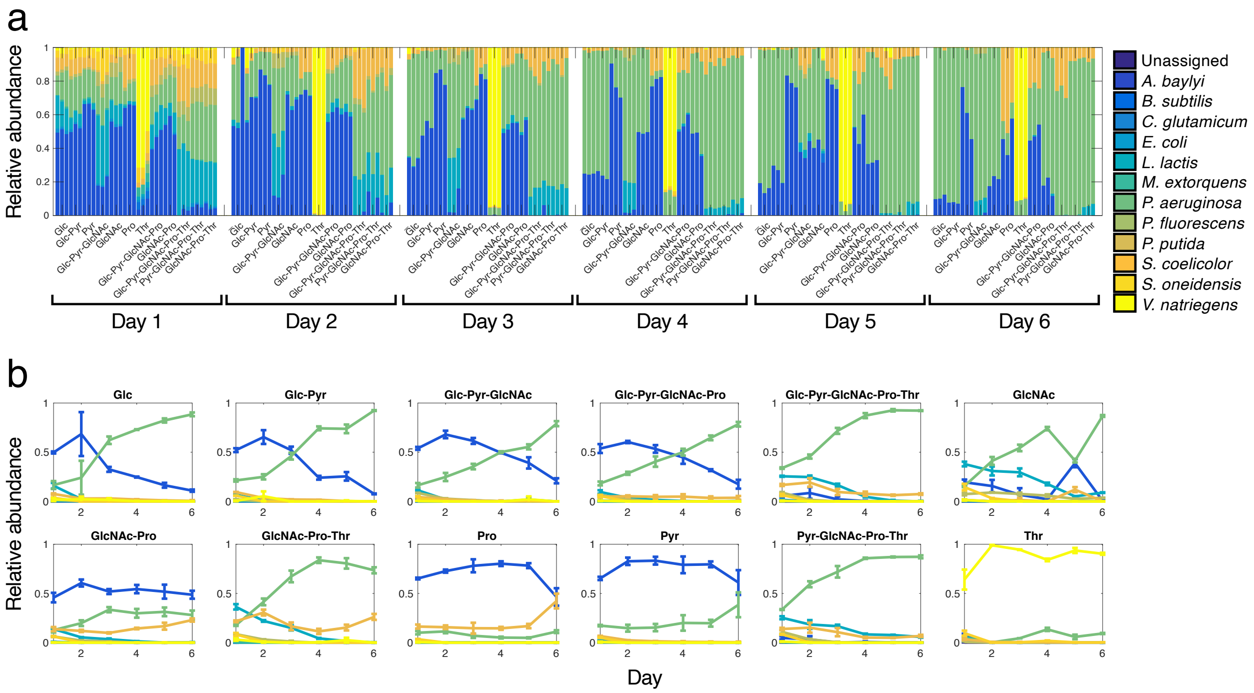

Supplementary Figure 4. Taxonomic data for 13-species community grown on five carbon sources (com13a, Supplementary Table 5): D-glucose (DGlc), pyruvate (Pyr), GlcNAc (GlcNAc), L-proline (Pro), and L-threonine (Thr). **(a).** Relative abundance plots of all replicates over time. **(b).** Species relative abundance trajectories over time. Data are represented as mean ± SD for three biological replicates.

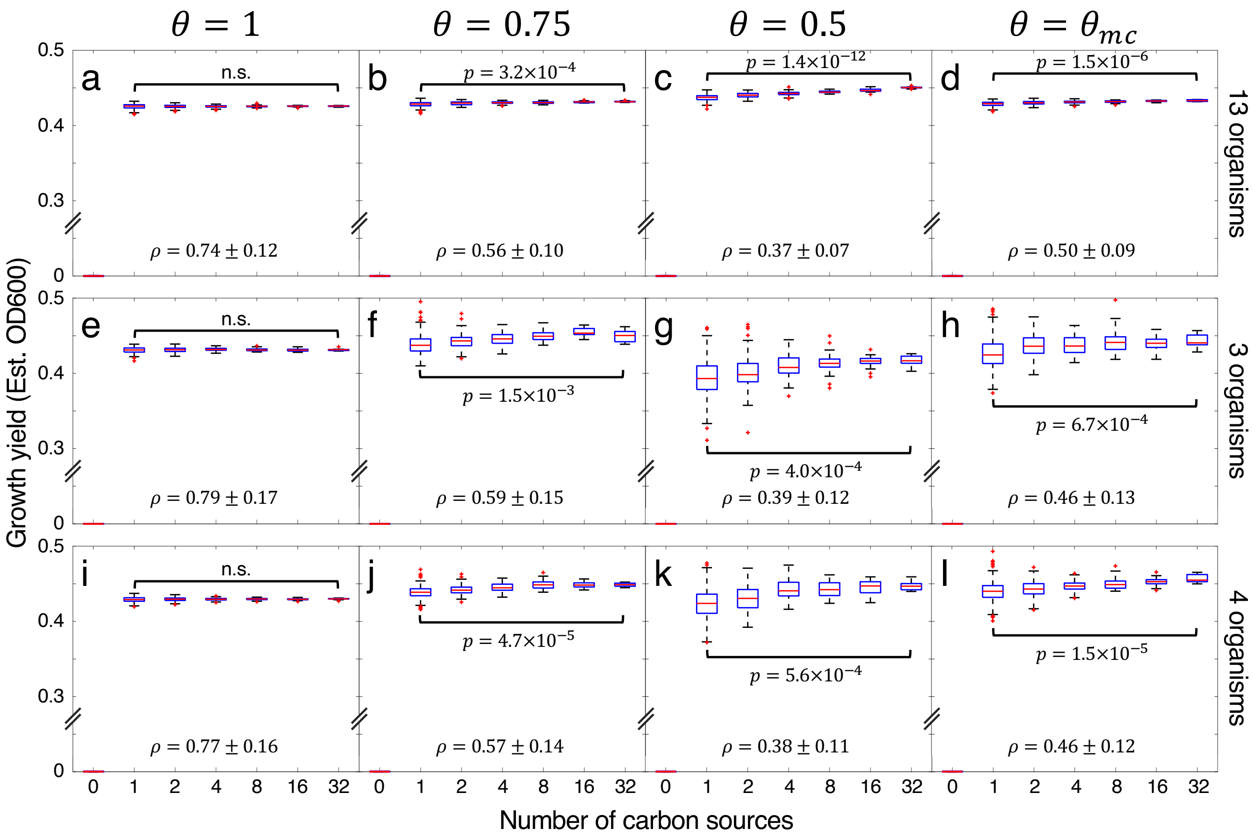

Supplementary Figure 5. Consumer resource model-predicted growth yields for simulated communities with different resource utilization capabilities. Communities with 13 **(a-d)**, 3 **(e-h)**, and 4 **(i-l)** initial organisms were simulated. For each organism i in a community, the rate at which it can consume a resource α is defined by the stoichiometric resource utilization matrix $C_{i\alpha}$ (Figure 1e, see Methods). In these simulations, a value in C can only be greater than zero if that organism's probability $P_{util}^{i,\alpha}$ of utilizing a given resource falls below a given threshold θ_i. Under each θ_i specified here, communities were simulated 50 times, for which the C and P_{util} values were randomly re-sampled. Decreasing θ thus allowed us to simulate diminishing degrees of organism-specific resource utilization capabilities, with lower θ values corresponding to more specialized organisms. Moreover, decreasing θ values also correspond to lower degrees of community niche overlap ρ (Supplementary Figure 10), allowing us to examine the effect of organism relatedness on community growth yield. Here, simulated yields for communities with $\theta_i = 1$ for all i **(a, e ,i)**, $\theta_i = 0.75$ for all i **(b, f, j)**, and $\theta_i = 0.5$ for all i **(c, g, k)** are shown. In **(d, h, l)**, we show simulated yields for communities where θ_i values correspond to the proportion of carbon sources mc able to be consumed by each organism i in our monoculture assays (Supplementary Figure 9). The red central mark indicates the median, the top and bottom box edges indicate the 25th and 75th percentiles, respectively, the whiskers extend to the most extreme points not considered outliers, and the red '+' symbols indicate outliers plotted individually. Paired one-sided t tests comparing the yield in 32 carbon sources to that in single carbon sources showed that no significant increases in yield occurred with increasing environmental complexity for $\theta_i = 1$, indicating yield additivity. However, significant increases in yield were observed for $\theta_i = 0.75$ and $\theta_i = 0.5$ in all communities, suggesting that the presence of more specialized organisms leads to lower probabilities of resource consumption in less complex environments. Lastly, the magnitude of yield increases was greater in our simulated 3- and. 4-species communities **(h, l)** than in our 13-species community **(d)**, which aligns with our experimental observations (Figure 2a-c). Final biomass values shown are after simulating 288 hours of community growth (corresponding to the full experimental timescale of com3, com4, and com13) on different combinations of resources. An OD600 of 1 is estimated at 8×10^8 CFU/mL.

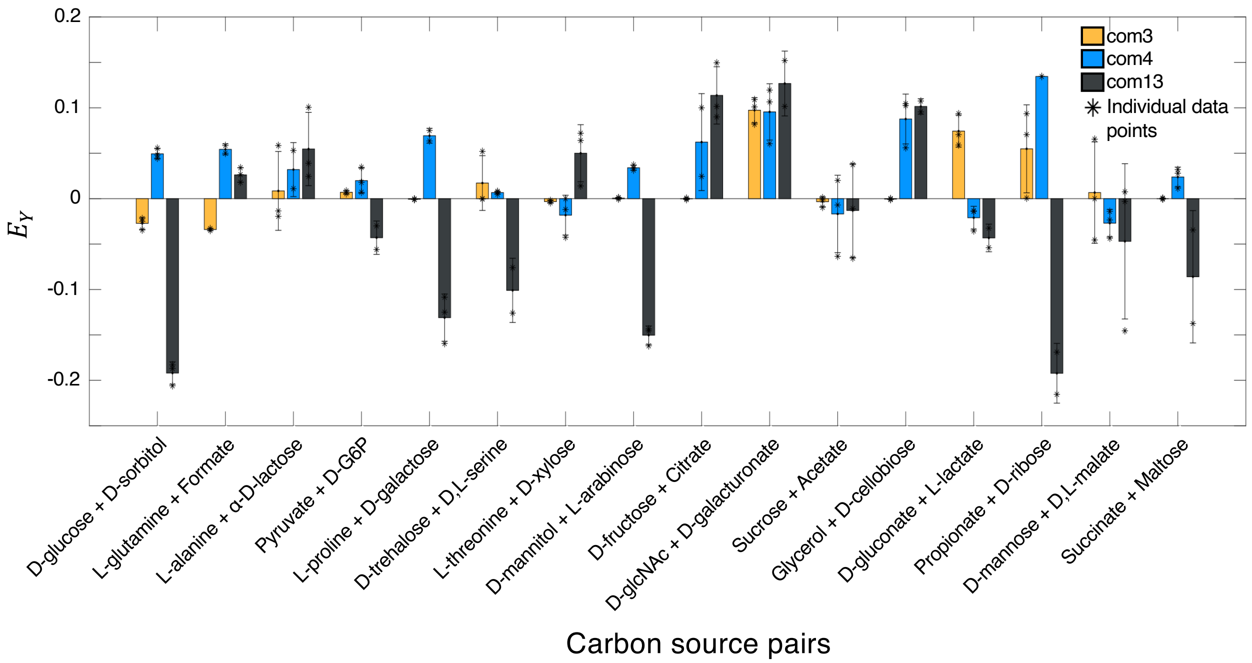

Supplementary Figure 6. Yield epistasis E_Y for com3, com4, and com13 between community yields on pairs of carbon sources and yields on the corresponding single carbon sources. Data are represented as mean ± SD across three biological replicates.

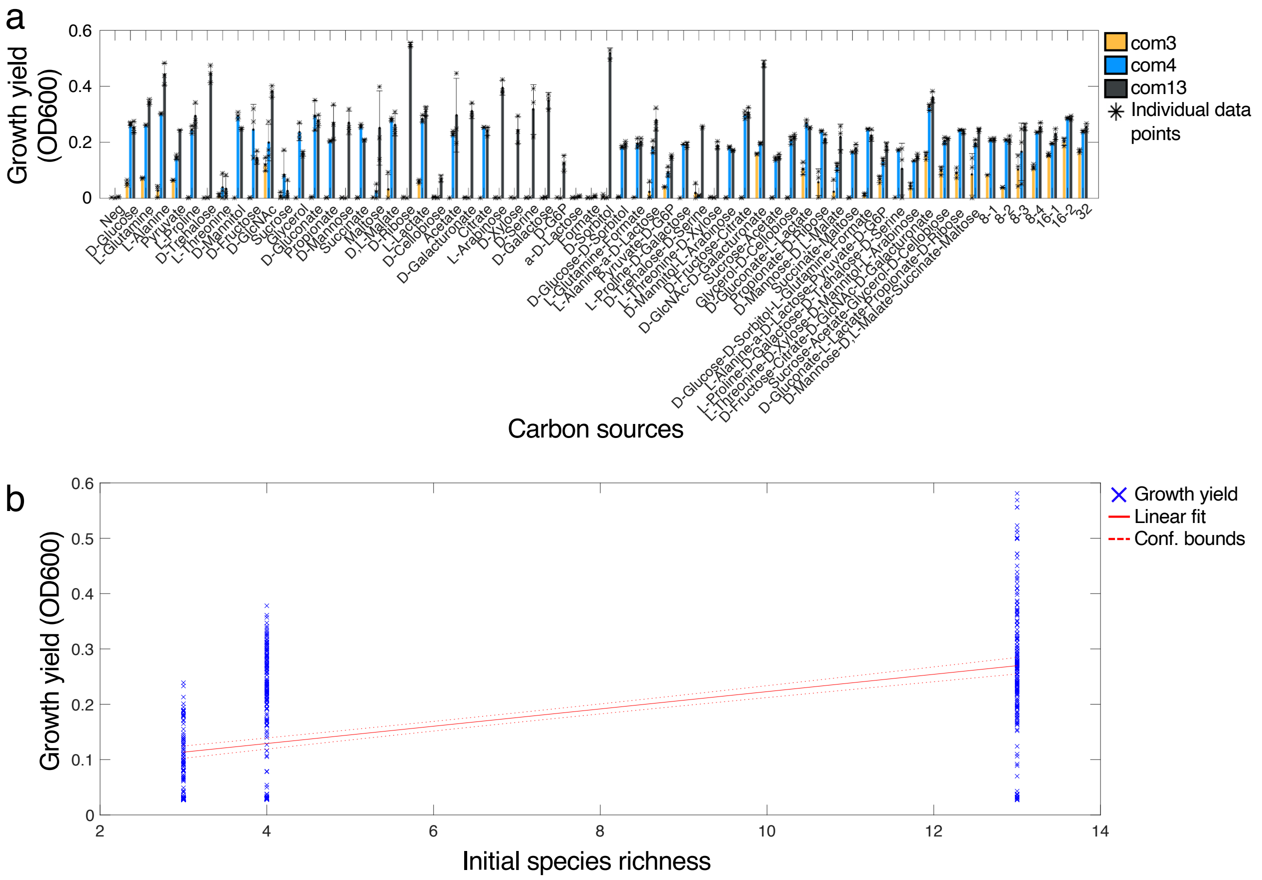

Supplementary Figure 7. Endpoint growth yields for multispecies communities com3, com4, and com13. **(a).** Final growth yields for communities by carbon source. Data are represented as mean ± SD across three biological replicates. **(b).** Statistical test quantifying increasing average community yields with higher initial species richness (one-way ANOVA *F*-statistic: 255, $p < 1 \times 10^{-47}$).

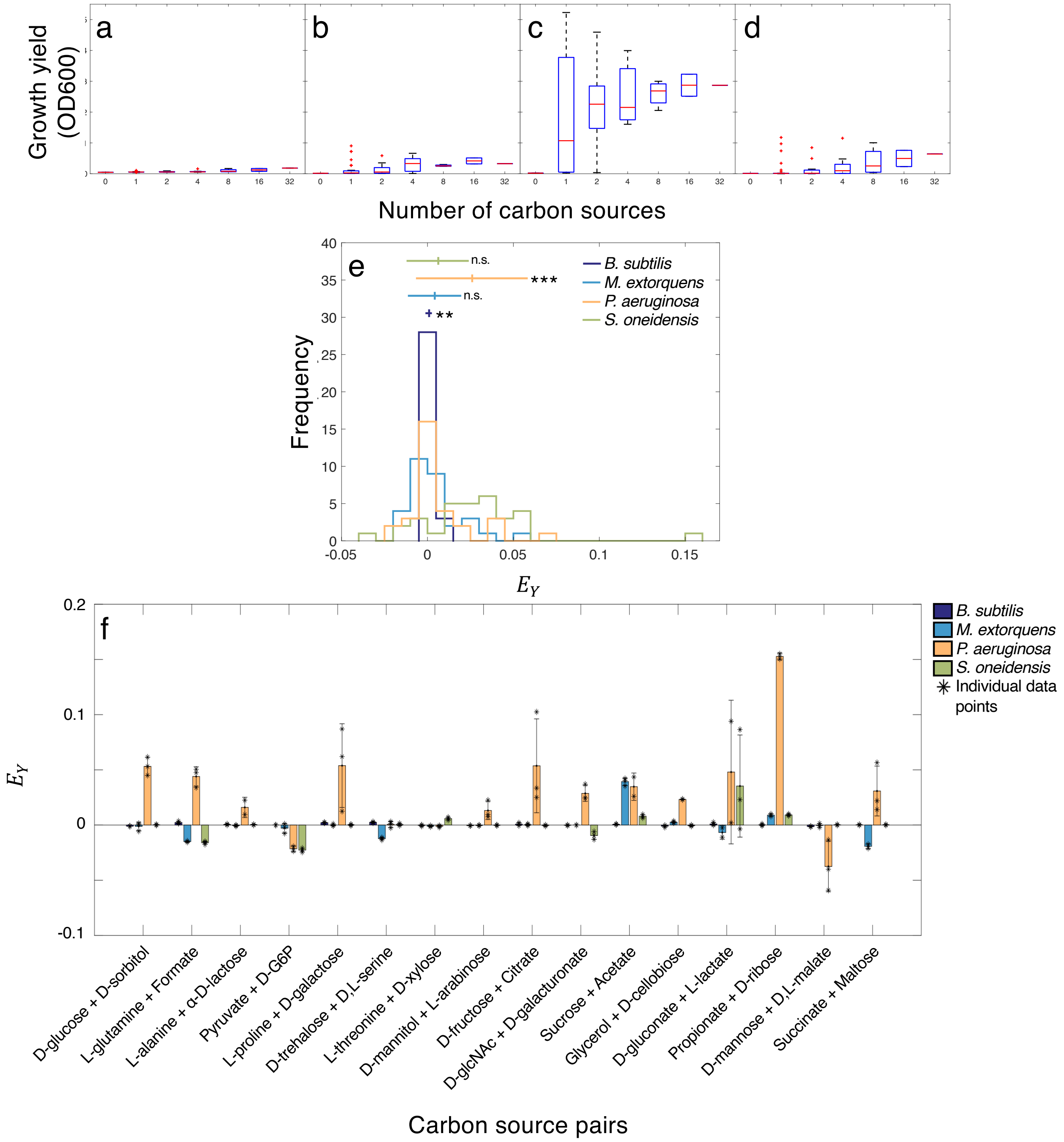

Supplementary Figure 8. Monoculture growth yields in combinatorial carbon sources for **(a)** *B. subtilis*, **(b)** *M. extorquens*, **(c)** *P. aeruginosa*, and **(d)** *S. oneidensis*. The red central mark indicates the median, the top and bottom box edges indicate the 25th and 75th percentiles, respectively, the whiskers extend to the most extreme points not considered outliers, and the red '+' symbols indicate outliers plotted individually. **(e)**. Distributions of yield epistasis E_Y for four organisms. Bars and notches indicate mean and standard deviation. Significance determined using a paired one-sided t test against a mean of zero and is indicated by (*) $p < 0.05$, (**) $p < 0.01$, and (***) $p < 0.001$. P-values for organisms are: 0.007 (*B. subtilis*), 0.122 (*M. extorquens*), 1.05e-4 (*P. aeruginosa*), and 0.056 (*S. oneidensis*). **(f).** Yield epistasis E_Y for four single organisms between pairs of carbon sources and corresponding single carbon sources. Data are represented as mean ± SD across three biological replicates.

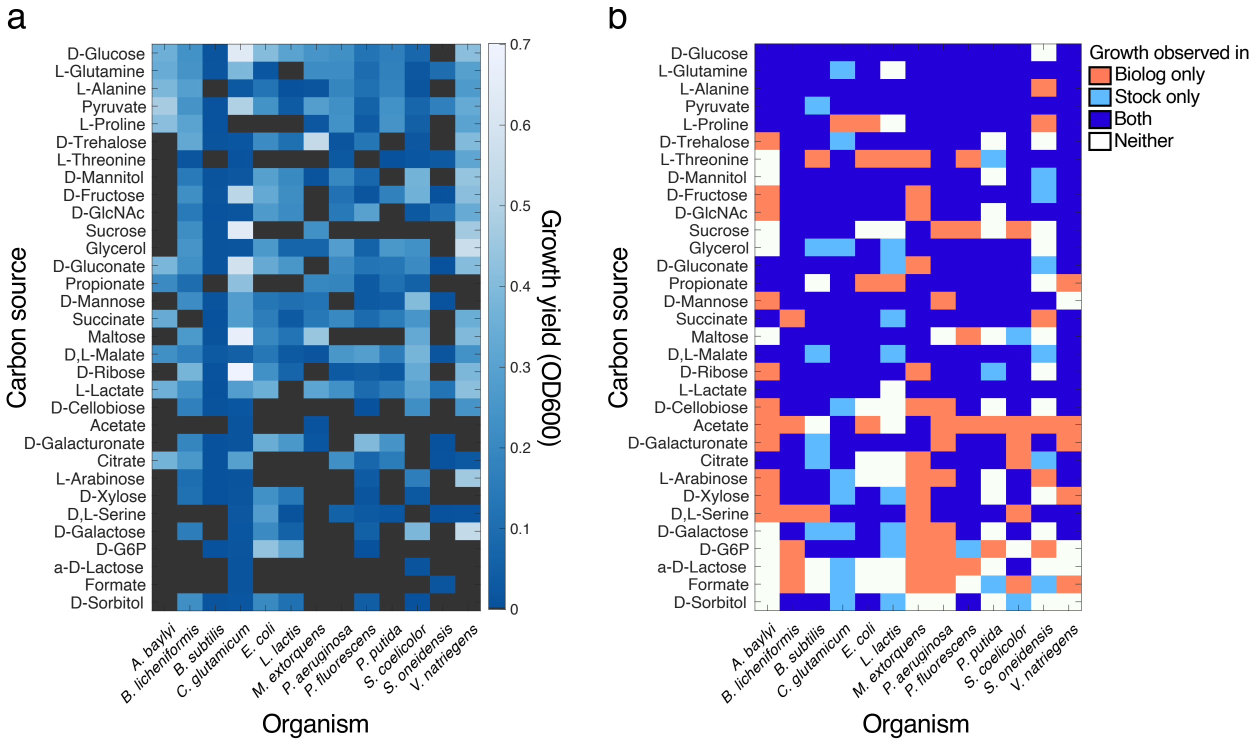

Supplementary Figure 9. Results of monoculture growth experiments and comparison to Biolog phenotypic assay for 32 selected carbon sources. **(a).** Average (3-biological replicate) species-specific growth yields on carbon sources prepared from stock solutions in M9 minimal medium after 48 hours. Raw OD600 values were corrected for liquid evaporation and a significance cutoff was applied to determine yields above the levels of the negative controls (see Methods). Carbon sources on which growth was not observed are marked in grey. **(b)**. Binary organism- and carbon source-specific comparisons of growth on carbon sources prepared from stock solutions vs. on carbon sources resuspended in Biolog plates (Supplementary Figure 2). Species-specific accuracies when comparing yields in stock solutions to growth in Biolog plates are: 65.6% for *A. baylyi*, 81.3% for *B. licheniformis*, 75.0% for *B. subtilis*, 65.6% for *C. glutamicum*, 87.5% for *E. coli*, 68.8% for *L. lactis*, 56.3% for *M. extorquens*, 68.8% for *P. aeruginos*a, 81.3% for *P. fluorescens*, 84.4% for *P. putida*, 75.0% for *S. coelicolor*, 62.5% for *S. oneidensis*, and 84.4% for *V. natriegens* for an overall average agreement of 73.6% between culturing methods.

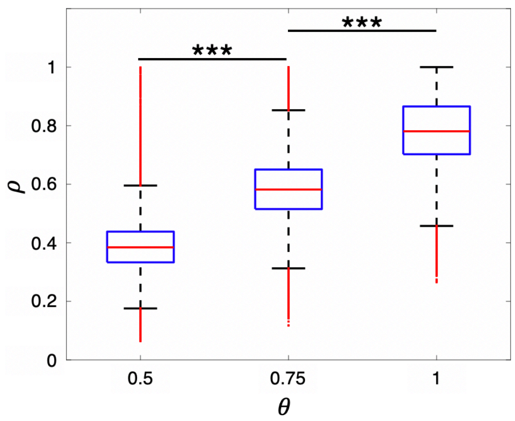

Supplementary Figure 10. Relationship between the fraction θ of resources usable by each organism and community niche overlap ρ in all 13-, 3-, and 4-species communities modeled using consumer resource models (Supplementary Figure 5, Supplementary Figure 19). For each θ, $n = 103{,}950$ communities (63 environmental conditions × (0,10) secreted metabolites × 50 random samplings × 3 community sizes (13, 3, 4 organisms)). Here, as with all boxplots, the red central mark indicates the median, the top and bottom box edges indicate the 25th and 75th percentiles, respectively, the whiskers extend to the most extreme points not considered outliers, and the red '+' symbols indicate outliers plotted individually. Paired one-sided t tests comparing distributions of ρ confirmed a positive relationship with θ (Significance indicated by (***) $p < 0.001$, $p < 10^{-20}$ for both comparisons).

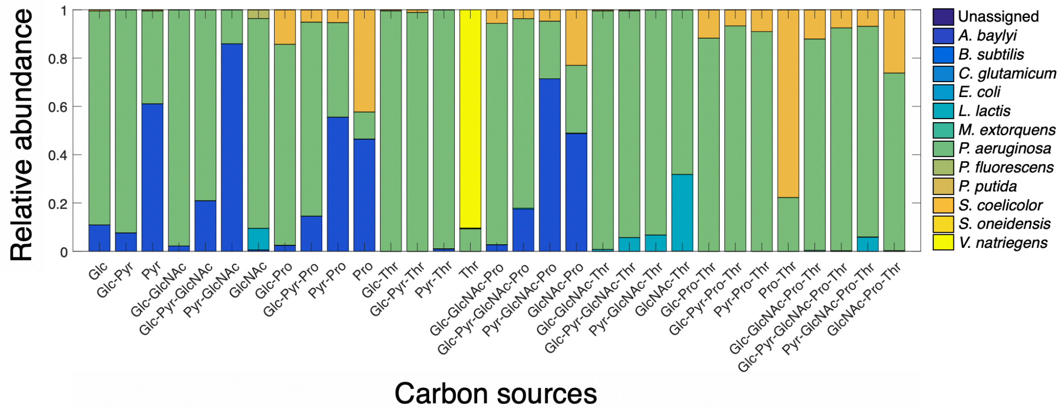

Supplementary Figure 11. Mean relative abundances (averaged over 3 biological replicates) for 13-species community grown on five carbon sources (com13a): D-glucose (DGlc), pyruvate (Pyr), GlcNAc (GlcNAc), L-proline (Pro), and L-threonine (Thr).

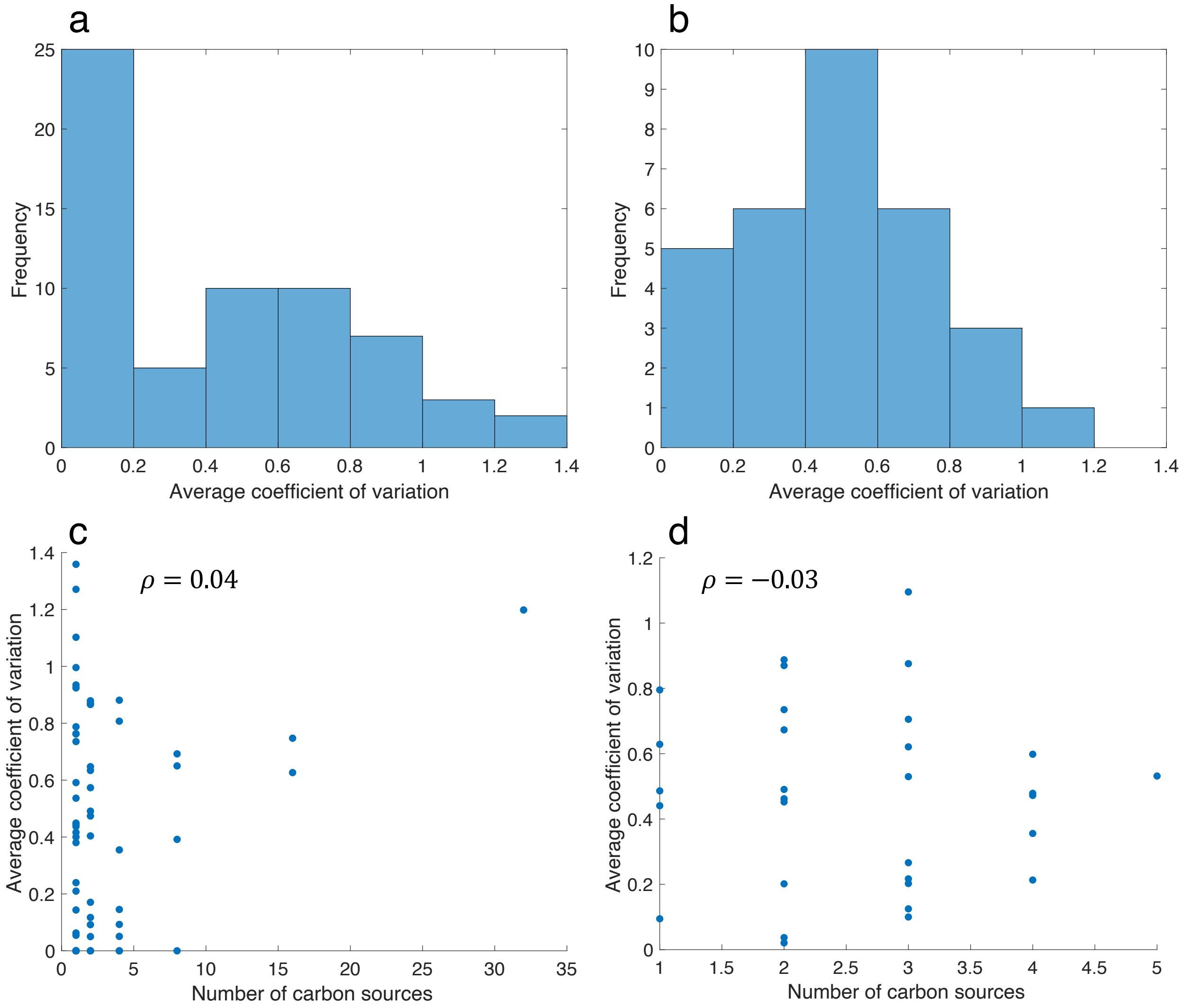

Supplementary Figure 12. Distributions of inter-replicate ($n = 3$) coefficients of variation for 13-species community grown on 32 carbon sources (com13, **a**) and 13-species community grown on 5 carbon sources (com13a, **b**). **(c, d).** Inter-replicate coefficients of variation vs. number of carbon sources for com13 **(c)** and com13a **(d)** with Spearman correlation coefficients ρ. No significant correlations were found between inter-replicate variability and environmental complexity for either community ($p = 0.73$ for com13 and 0.86 for com13a).

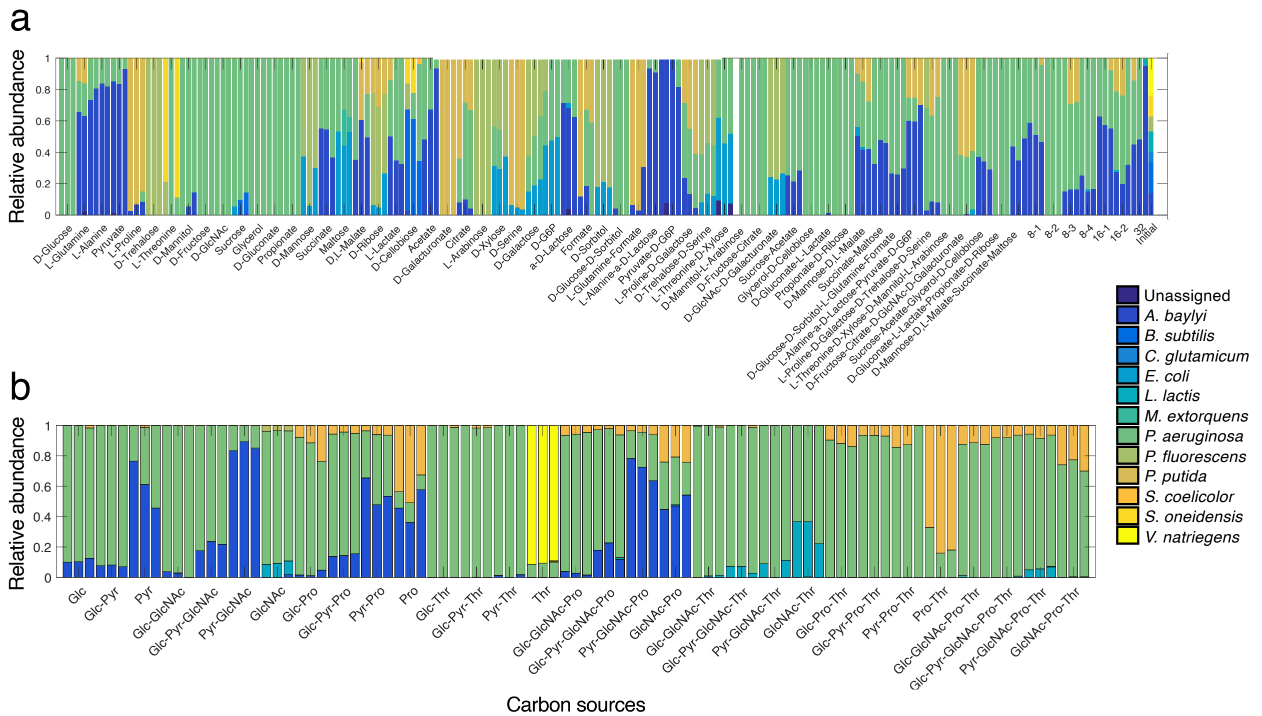

Supplementary Figure 13. Endpoint taxonomic distributions over all biological replicates for 13-species community grown on 32 carbon sources (com13, **a**) and 13-species community grown on 5 carbon sources (com13a, **b**). We encountered general consistency in community composition between comparable conditions of the two 13-species experiments. The D-glucose, pyruvate, and D-GlcNAc conditions had the same dominant organisms in both experiments (*P. aeruginosa*, *A. baylyi*, and *P. aeruginosa*, respectively), and the L-proline condition was composed of *A. baylyi*, *P. aeruginosa*, and a third organism in both experiments with only the identity of the third organism being different (*P. putida* in com13 and *S. coelicolor* in com13a). Nonetheless, in com13, L- threonine resulted in a dominance of *S. oneidensis* in two of the replicates and of *P. aeruginosa* in one replicate, while com13a resulted in dominance of *V. natriegens* in all three replicates. However, com13 grew very minimally in L-threonine (OD600 0.03 ± 0.04) in comparison to com13a (0.15 ± 0.02), in addition to having a very high inter-replicate coefficient of variation for this condition (1.36), suggesting that it is an outlier.

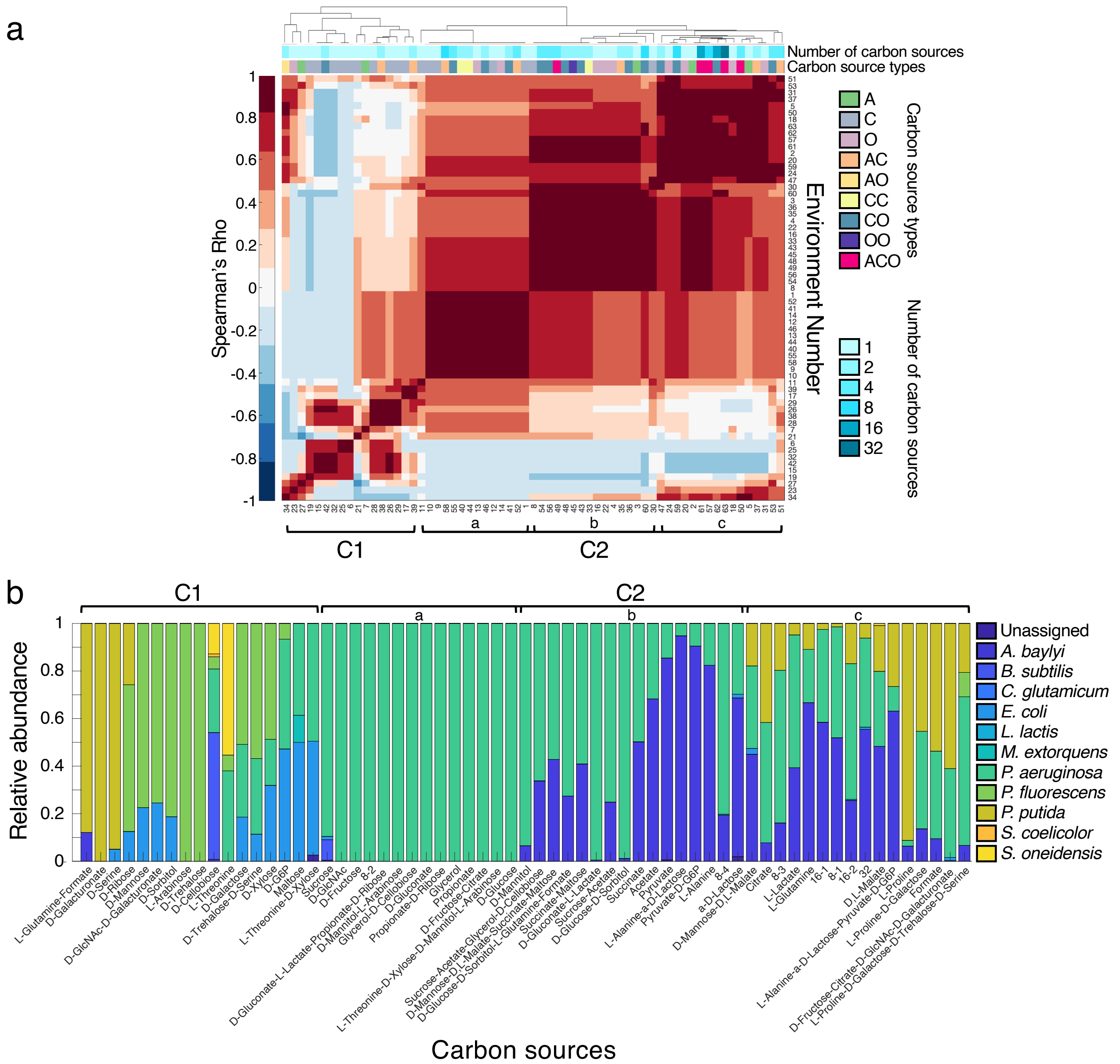

Supplementary Figure 14. Hierarchical clustering of 63 carbon source combinations according to Spearman correlations between community taxonomic distributions. **(a).** Clustering diagram. Environments are numbered according to Supplementary Table 3. **(b).** Mean (across 3 biological replicates) species relative abundances ordered according to clusters. Environments with more than two carbon sources are abbreviated (e.g. condition 4-1 contains the carbon sources in the first two 2-carbon source conditions, etc.). Compositions of the 63 environments are provided in Supplementary Table 3. Clusters C1 and C2a-c are designated according to higher-level branches. C1 contains 17 conditions and C2 contains the remaining 46. Conditions that were clustered closely in C1 included D-mannose and D-glcNAc + D-galacturonate (conditions 15 and 42), which displayed almost equal distributions of *E. coli* and *P. fluorescens*, as well as conditions 26, 28, 29, and 38 (D-xylose, D-galactose, D-G6P, and D-trehalose + D,L-serine), which displayed distributions of *E. coli*, *P. aeruginosa*, and *P. fluorescens*. Subcluster C2a contains a variety of carbon source combinations ranging from one to 8 carbon sources. Despite this variability, these environments all resulted in communities that were dominated by *P. aeruginosa*. Subcluster C2b was mainly represented by environments containing organic acids, which yielded communities composed of *A. baylyi* and *P. aeruginosa*. Subcluster C2c contained the most environmentally-complex conditions (with both 16-carbon source and the 32-carbon source condition) and displayed some of the most pronounced differences when compared to those in other clusters. In particular, conditions 15, 32, and 42 (D-mannose, D-sorbitol, and D-glcNAc + D-galacturonate) displayed the most dissimilar community compositions compared to subcluster C2c.

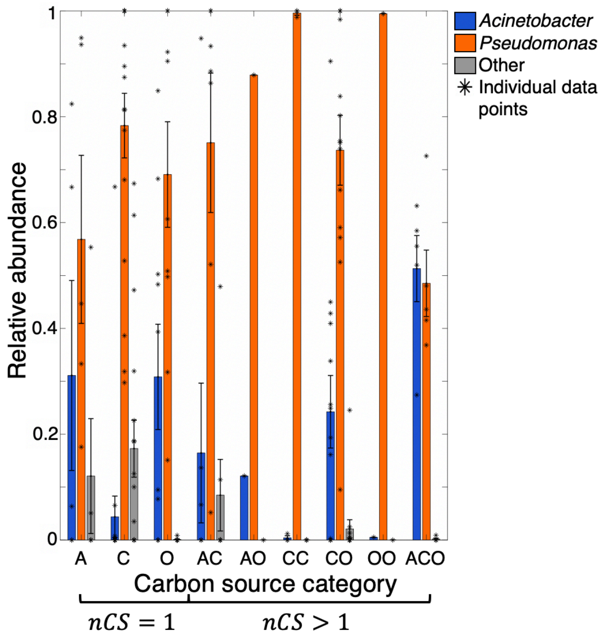

Supplementary Figure 15. Relative abundances of organism groupings (*Acinetobacter*, *Pseudomonas*, and 'Other' for all other organisms) that emerged from hierarchical clustering according to carbon source type (A: amino acid, C: carbohydrate, O: organic acid). Single-carbon source conditions ($nCS = 1$) contain only one type, while multiple-carbon source conditions ($nCS > 1$) contain at least one of the types shown. For example, the L-glutamine condition would be categorized under 'A' for amino acid, while the L-glutamine + formate condition would be categorized as 'AO' as it contains both an amino acid and an organic acid. Carbon source-specific type designations are provided in Supplementary Table 2. Sample sizes for each carbon source type are: A: 5, C: 17, O: 10, AC: 7, AO: 1, CC: 3, CO: 14, OO: 1, ACO: 5. Data are represented as mean ± SD.

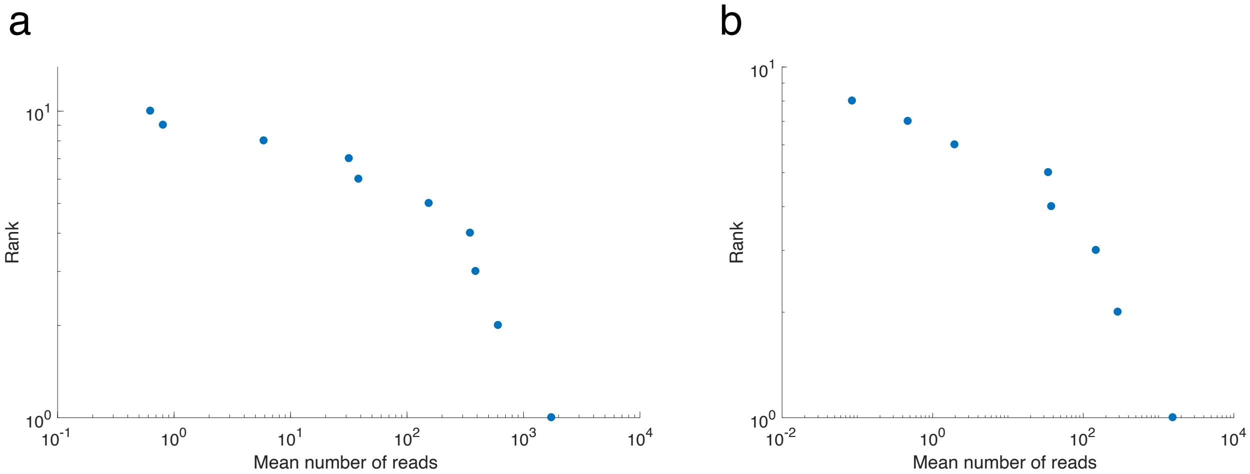

Supplementary Figure 16. Rank-abundance plots for 13-species community grown on 32 carbon sources (com13, **a**) and 13-species community grown on 5 carbon sources (com13a, **b**). Each point represents the mean (across 3 biological replicates) number of reads for an individual organism. These relationships displayed decay patterns separated at characteristic scales of approximately 10^2 reads, resembling double-power law relationships previously observed in a variety of natural ecosystems orders of magnitude more complex than our model communities[16–18]. Despite difference in scale, this rank-abundance relationship suggests fundamental structural similarities in community composition across experimental systems. Moreover, this similarity extends to the scaling and prevalence of very low-abundance taxa, indicating the abundances of these community members are accurately represented within our populations.

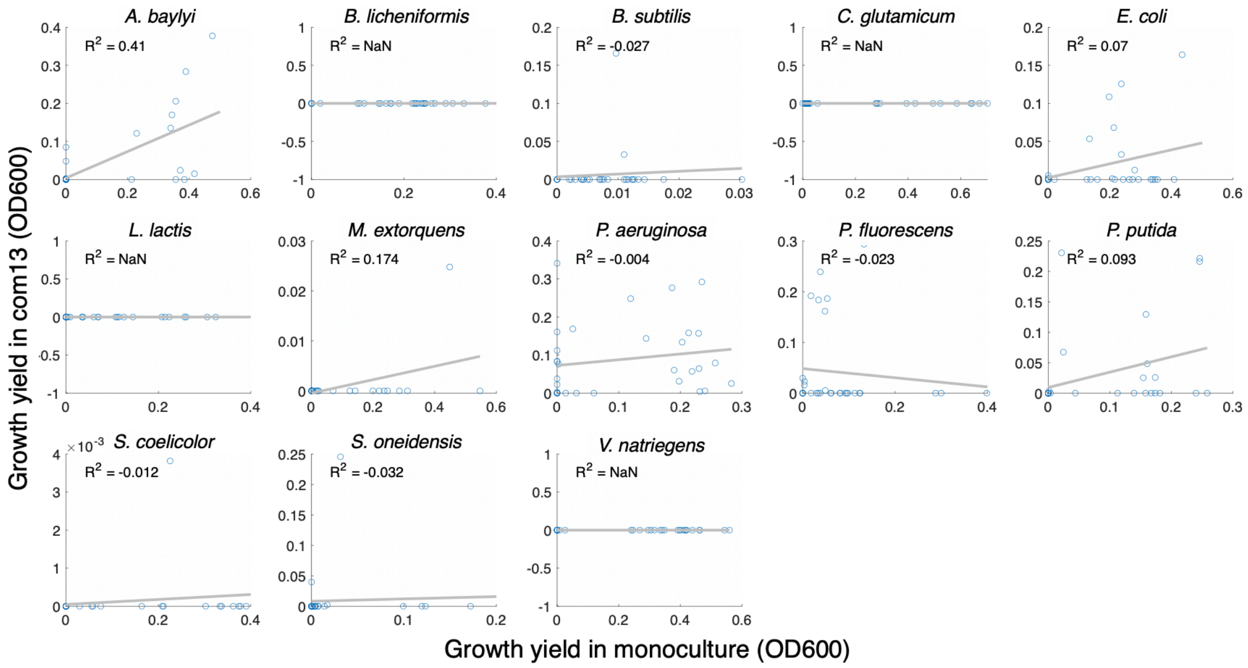

Supplementary Figure 17. Mean (across 3 biological replicates) organism-specific yields in 13-species community grown on 32 carbon sources (com13) vs. in monoculture, with best-fit lines calculated using ordinary least squares regression.

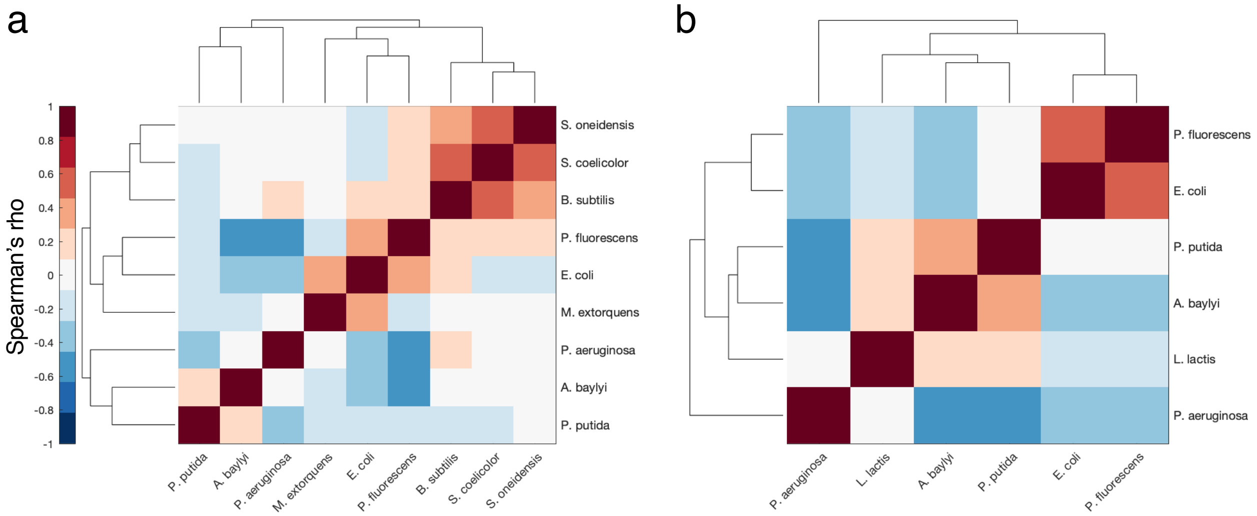

Supplementary Figure 18. Hierarchical clustering of Spearman correlations between organism relative abundances across com13 environments (Supplementary Table 3). **(a).** Clustering of species-species correlations across single-carbon source conditions. We found that the overall structure of the species-species clusters in mixed cultures was dramatically different from that of the monocultures (Supplementary Figure 2d), with much lower degrees of interspecies similarities. In com13, higher degrees of similarity were observed between *B. subtilis*, *S. coelicolor*, and *S. oneidensis*, likely due to their co-occurrence in D-cellobiose. This similarity was not observed in the monoculture data, in which *B. subtilis* correlated more strongly with organisms that did not remain in com13. The species-species clustering of com13 also highlighted the profound dissimilarities between *P. fluorescens* and both *A baylyi* and *P. aeruginosa*. These anticorrelations contrast with the monoculture data, as *P. fluorescens* had displayed relatively high degrees of similarity with both organisms. This difference further clarifies the competitive effects observed between *P. fluorescens* and these two organisms in a community setting, which leads to exclusion of specific organisms despite their ability to utilize the provided carbon sources. **(b).** Clustering of species-species correlations across multiple-carbon source conditions. We observed that the similarities between *E. coli* and *P. fluorescens* were more pronounced than in the single-carbon source conditions, reflecting the ability of these two organisms to coexist in across different environments (e.g. D-trehalose + D-serine and D-glcNAc + D-galacturonate). Despite also having the ability to coexist in more complex environments, the correlations between *A. baylyi* and *P. aeruginosa* decreased in the multiple-carbon source conditions. In fact, *P. aeruginosa* was found to be the most dissimilar organism in these environments, likely due to its ability to exclude all other organisms in many conditions.

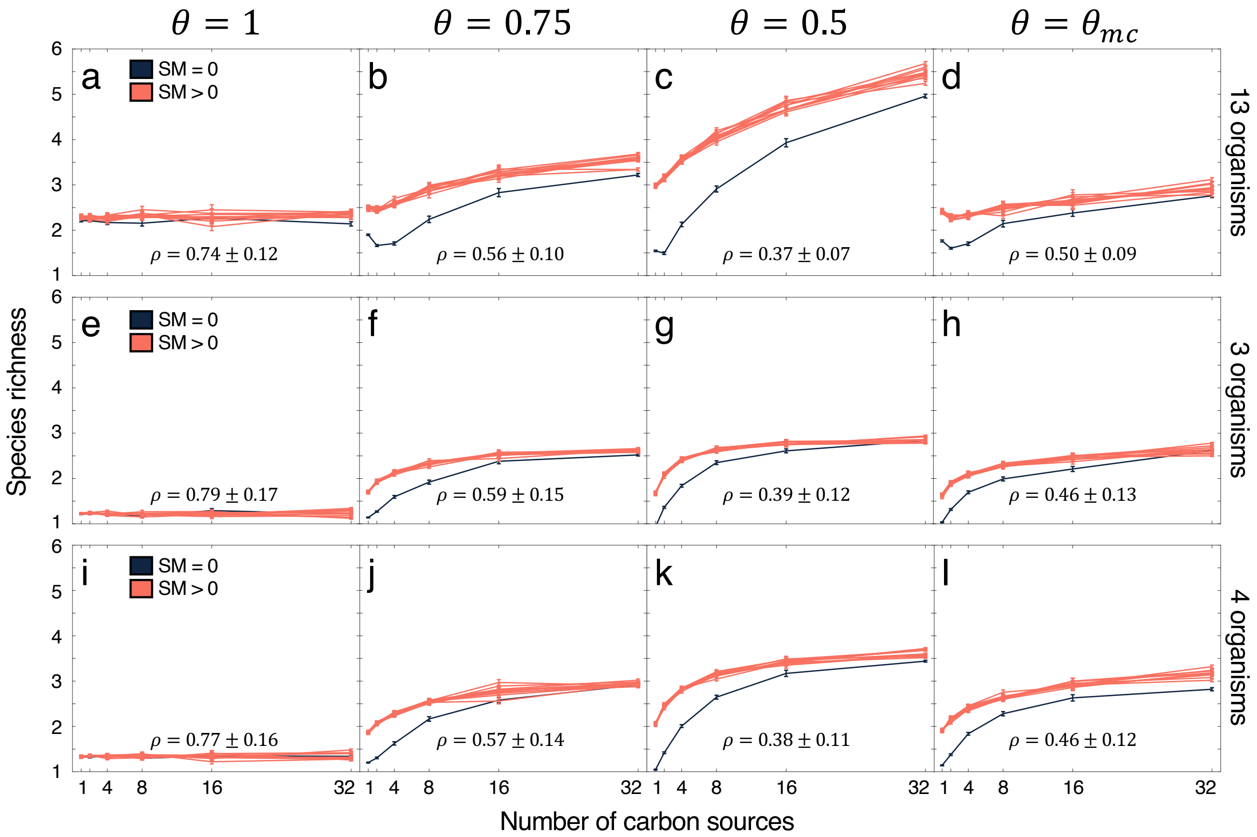

Supplementary Figure 19. Consumer resource model-predicted species richness values for simulated communities with different resource utilization capabilities. These communities, whose growth yields are shown in Supplementary Figure 5, were modeled with varying resource utilization capabilities (denoted by θ, see Methods). Decreasing θ values also correspond to lower degrees of community niche overlap ρ (Supplementary Figure 10), allowing us to examine the effect of organism relatedness on taxonomic diversity. Under each θ_i specified here, communities were simulated 50 times, in which the organisms' resource utilization matrices were resampled. We also simulated communities with either zero (black lines) or between 1 and 10 (orange lines) unique available secreted metabolites (SM) in order to assess the effects of metabolic exchange on diversity. Here, simulated final species richness values for communities with $\theta_i = 1$ for all i **(a, e ,i)**, $\theta_i = 0.75$ for all i **(b, f, j)**, and $\theta_i = 0.5$ for all i **(c, g, k)** are shown. In **(d, h, l)**, we show simulated final species richness values for communities where θ_i values correspond to the proportion of carbon sources mc able to be consumed by each organism i in our monoculture assays (Supplementary Figure 9). We observed an inverse relationship between niche overlap and increasing taxonomic diversity, indicating that interspecies competition has a dampening effect on organism coexistence. Moreover, our results suggest that the availability (but not necessarily quantity) of secreted metabolites can mitigate the effects of resource competition. Final species richness values shown are after simulating 288 hours of community growth (corresponding to the full experimental timescale of com3, com4, and com13) on different combinations of resources. Data are represented as mean ± SEM.

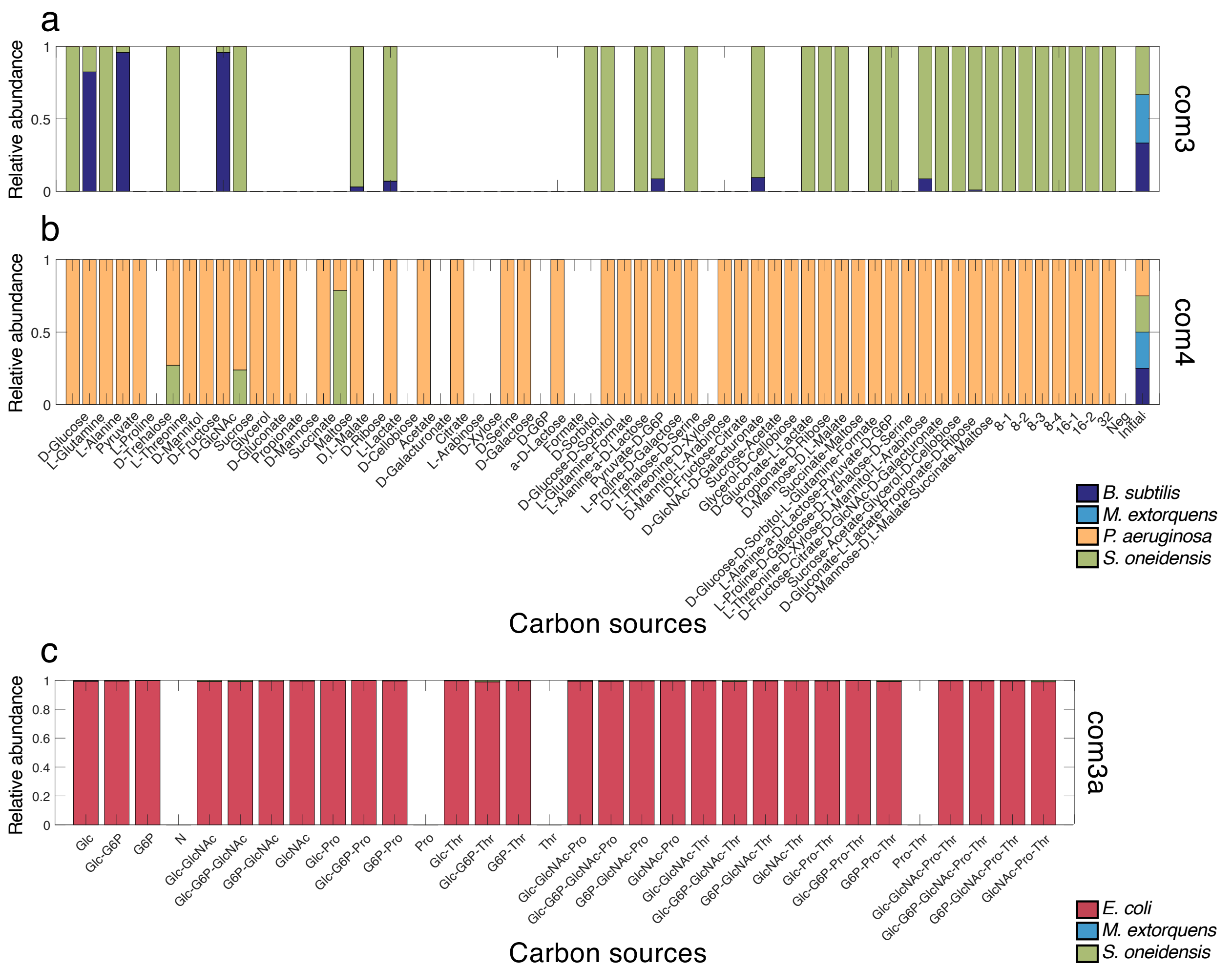

Supplementary Figure 20. Mean (across 3 biological replicates) endpoint species relative abundances for multispecies communities com3 **(a)** and com4 **(b)**, and com3a **(c)**. *P. aeruginosa* was most often dominant in com4, outcompeting the other community members in 52 cases. In addition to the dominance of *P. aeruginosa* in com4, we noticed that *S. oneidensis* was dramatically overrepresented in our com3 experiment. This distribution was striking, as we expected *M. extorquens* to be most often dominant given its wider breadth of resource utilization capabilities in monoculture (Supplementary Figure 2a). **(c)**. An additional experiment containing *E. coli*, *M. extorquens*, and *S. oneidensis* (com3a, Supplementary Table 5) similarly highlighted the ability of a single organisms to overtake small communities. Relative abundances for com3 and com4 are adjusted based on a calibration of CFU counts to equal OD600 values. Unabbreviated environmental compositions for com3 and com4 are outlined in Supplementary Table 3. Unique conditions for com3a are: D-glucose (DGlc), pyruvate (Pyr), GlcNAc (GlcNAc), L-proline (Pro), L-threonine (Thr), and a no-carbon negative control (N). Missing bars indicate no growth in the specified condition.

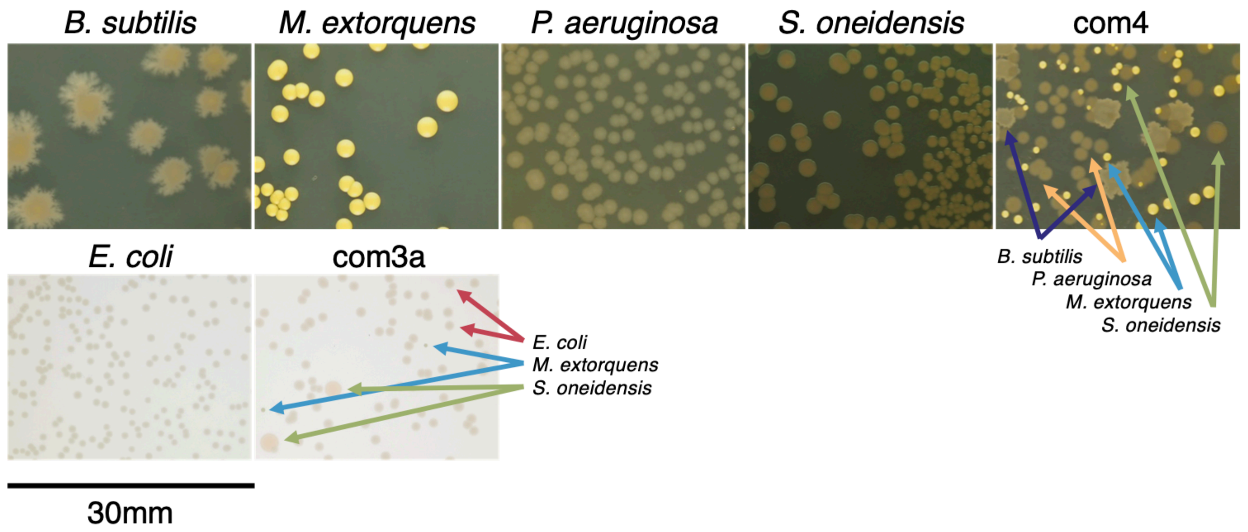

Supplementary Figure 21. Differentiable colony morphologies for organisms assayed by agar plating. Colonies shown are representatives of three biological replicates plated for each species or community on LB agar (see Methods).

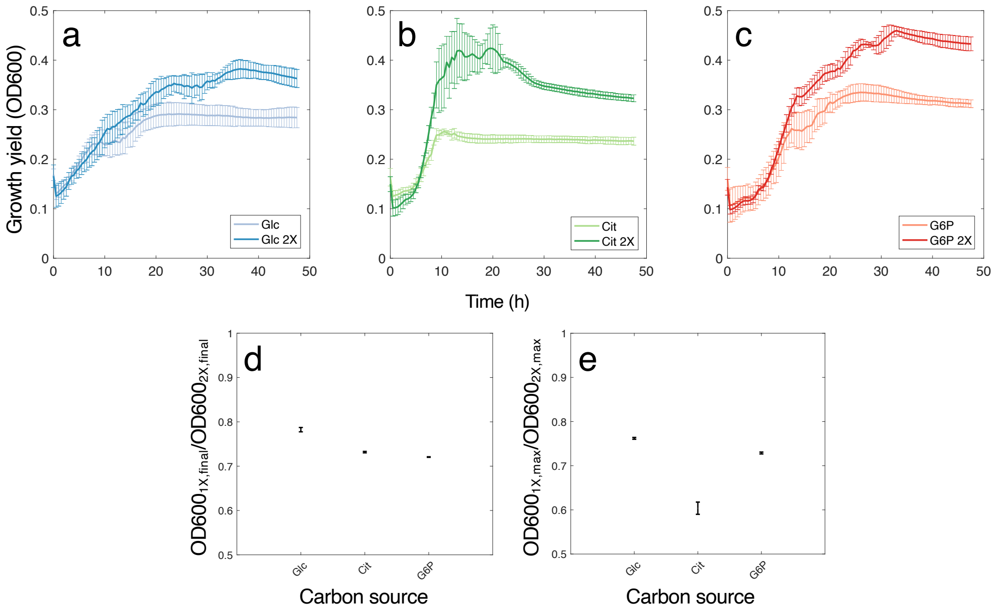

Supplementary Figure 22. Analysis of community growth yields in single (25 mM C) and double (50 mM C) concentrations of carbon sources. **(a-c).** Growth trajectories for 14-species community (com14) in two concentrations of D-glucose (Glc, **a**), citrate (Cit, **b**), and glucose-6-phosphate (G6P, **c**). **(d).** Ratio of community biomass values between single- and double-carbon source concentrations at endpoint **(d)** and for maximum OD values **(e)**. As the amount of carbon source was doubled, we expected a ratio of biomass at of 0.5 between the two concentrations. However, the observed average ratio was 0.74 ± 0.03, suggesting that some organisms grow more efficiently on diminished resource concentrations. Data are represented as mean ± SD across three biological replicates.

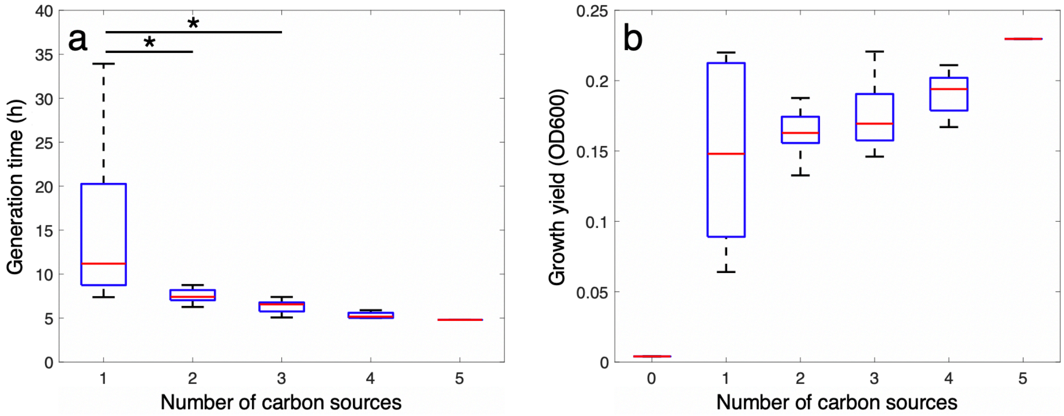

Supplementary Figure 23. Growth phenotype for 13-species community com13a. **(a).** Generation time vs. number of carbon sources for 13-species community grown on five carbon sources (com13a, Supplementary Table 5). Generation time was calculated by obtaining the maximum slope in biomass (Supplementary Figure 3b) using a moving window encompassing 5 hours. Significance is calculated by comparing generation times between single- and multiple-carbon source conditions and is indicated by (*) p < 0.05 (paired one-sided t test $p = 0.03$ for 1 vs. 2 carbon sources, 0.02 for 2 vs. 3 carbon sources, 0.52 for 3 vs. 4 carbon sources, and 0.33 for 4 vs. 5 carbon sources). **(b).** Growth yields grouped by the number of carbon sources in each environment at the end of the experiment (144h). For complete description of sample size see Supplementary Table 4. No significant increase in yield with environmental complexity was detected (paired one-sided t test $p = 0.49$ for 1 vs. 2 carbon sources, 0.18 for 2 vs. 3 carbon sources, 0.20 for 3 vs. 4 carbon sources, and 0.10 for 4 vs. 5 carbon sources). The red central mark indicates the median, the top and bottom box edges indicate the 25th and 75th percentiles, respectively, the whiskers extend to the most extreme points not considered outliers, and the red '+' symbols indicate outliers plotted individually.

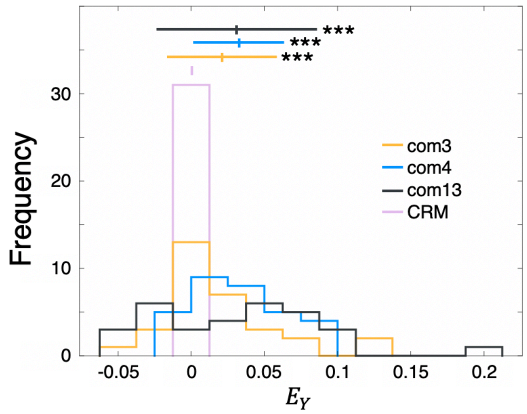

Supplementary Figure 24. Yield epistasis E_Y for com3, com4, and com13 after the first 48 hours of growth. P-values for each community are: 0.002 (com3), 1.1e^{-7} (com4), and 0.001 (com13) compared against the CRM distribution using a one-sided t test. Upper bars denote mean and standard deviation.

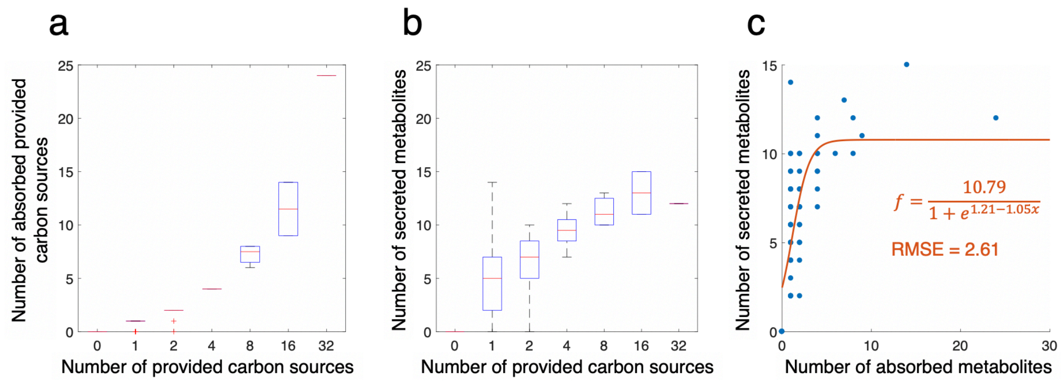

Supplementary Figure 25. Flux balance analysis-predicted number of absorbed and secreted metabolites for 4-species community made up of *B. subtilis*, *M. extorquens*, *P. aeruginosa*, and *S. oneidensis* in combinations of up to 32 carbon sources (Sample sizes are outlined in Supplementary Table 3). **(a).** Number of carbon sources taken up by any of the four organisms vs. number of provided carbon sources. **(b).** Number of secreted metabolites secreted by any of the four organisms vs. the number of provided carbon sources. In **a** and **b**, the red central mark indicates the median, the top and bottom box edges indicate the 25th and 75th percentiles, respectively, the whiskers extend to the most extreme points not considered outliers, and the red '+' symbols indicate outliers plotted individually. **(c).** Relationship between number of secreted metabolites and absorbed metabolites (provided carbon sources plus available secreted metabolites). A logistic function provided the best fit for this data, indicating a sharp rise followed by a plateau in the number of unique secreted metabolites.

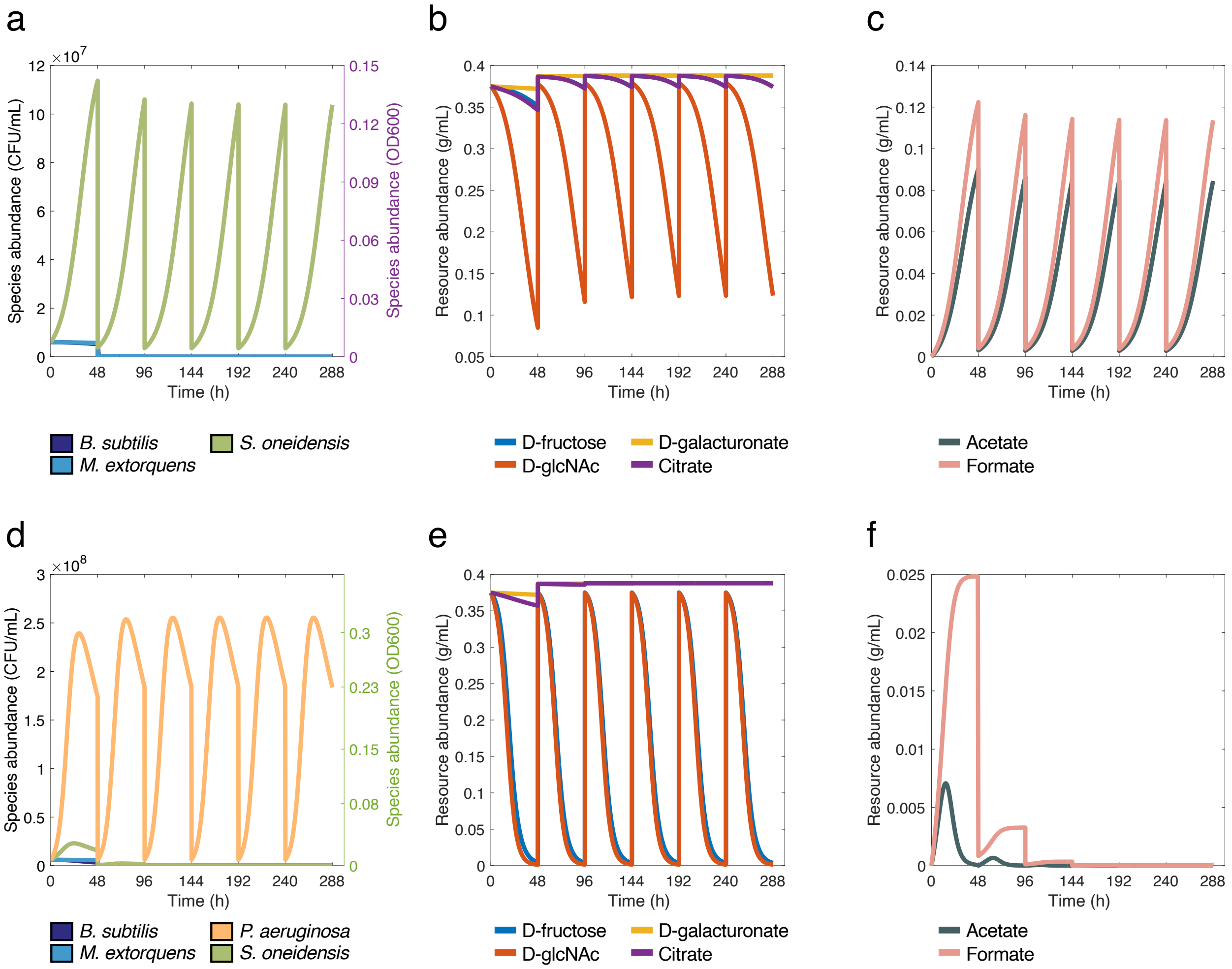

Supplementary Figure 26. Example consumer resource model-predicted growth phenotypes for com3 **(a-c)** and com4 **(b-f)** grown on D-fructose, citrate, D-glcNAc, and D-galacturonate (environment 53, Supplementary Table 3). Organism resource utilization efficiencies are parameterized using monoculture growth data (Supplementary Figure 9a), and metabolite secretion is parameterized using flux-balance analysis (Supplementary Methods, Supplementary Note 3) Community compositions and yields recapitulate those observed experimentally (Supplementary Figure 9a, Supplementary Figure 7a, Supplementary Figure 20a, b) and reveal environment-specific resource utilization patterns.

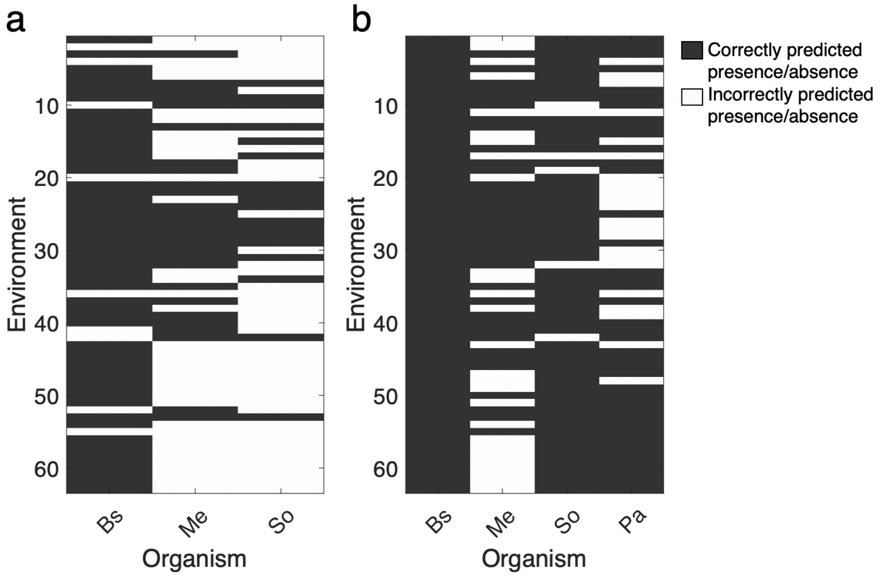

Supplementary Figure 27. Consumer resource model predictions compared to com3 and com4 experiments. **(a, b).** Environment-by-environment comparison of model-predicted and experimentally-observed presence/absence of individual organisms in com3 **(a)** and com4 **(b)**. Species-specific accuracies are: 85.7% for *B. subtilis* (Bs), 42.7% for *M. extorquens* (Me), and 28.6% for *S. oneidensis* (So) in com3, and 100% for Bs, 57.1% for Me, 90.5% for So, and 65.1% for *P. aeruginosa* (Pa) in com4. In both cases, a large portion of the prediction error is brought about by an overestimation of the persistence of *M. extorquens* in mixed culture. This is likely due to its measured ability to metabolize a variety of resources more efficiently than *S. oneidensis* and *P. aeruginosa* (Supplementary Figure 9), which formed the basis for the model parametrization. While this parametrization was sufficient to recapitulate the competitive advantages of *P. aeruginosa* in most cases (as well as the disadvantages of *B. subtilis* in com3 and com4 and *S. oneidensis* in com4), its inability to correctly predict the low abundance of *M. extorquens* in mixed culture suggests the presence of non-metabolic competitive effects not fully captured by the model.

Supplementary Tables

Supplementary Table 1. Complete list of organisms used in experiments.

Organism name	Strain	ATCC	Temp. (˚C)	Flux Balance Model
Acinetobacter baylyi	ADP1	33305	37	[19]
Bacillus licheniformis	46	14580	37	
Bacillus subtilis	168	23857	37	[8]
Corynebacterium glutamicum	534	13032	37	
Escherichia coli	MG1655	25922	37	[20]
Lactococcus lactis	MG1363	19257	37	[21]
Methylobacterium extorquens	AM1	43645	30	[9]
Pseudomonas aeruginosa	PA01	9027	37	[10]
Pseudomonas fluorescens	28/5	13525	26	
Pseudomonas putida	KT2440	47054	26	[22]
Streptomyces coelicolor	A3(2)	23899	28	[23]
Shewanella oneidensis	MR-1	700550	30	[11]
Salmonella enterica serovar Typhimurium	LT2	27106	37	[24]
Streptococcus thermophilus	LMG18311	BAA-250	37	
Vibrio natriegens	111	14048	26	

Supplementary Table 2. List and ontology of 95 carbon sources in Biolog PM1 plate.

Carbon source name	Class 1	Class 2	Class 3
1,2-Propanediol	Sugar alcohols	Monosaccharides	Carbohydrates
2-Aminoethanol	Sugar alcohols	Monosaccharides	Carbohydrates
2-Deoxyadenosine	Ribonucleosides	Nucleosides	Nucleic acids
a-D-Lactose	Disaccharides	Disaccharides	Carbohydrates
a-Hydroxyglutarate-g-lactone	Esters	Carboxylic acids	Organic acids
a-Hydroxybutyrate	2-Oxocarboxylic acids	Carboxylic acids	Organic acids
a-Ketobutyrate	2-Oxocarboxylic acids	Carboxylic acids	Organic acids
a-Ketoglutarate	2-Oxocarboxylic acids	Carboxylic acids	Organic acids
a-Methylgalactoside	Carbohydrate derivatives	Glycans	Carbohydrates
Acetate	Monocarboxylic acids	Carboxylic acids	Organic acids
Acetoacetate	3-Oxocarboxylic acids	Carboxylic acids	Organic acids
Adenosine	Ribonucleosides	Nucleosides	Nucleic acids
Adonitol	Sugar alcohols	Monosaccharides	Carbohydrates
b-Me-D-glucoside	Carbohydrate derivatives	Glycans	Carbohydrates
Bromosuccinate	Monocarboxylic acids	Carboxylic acids	Organic acids
Citrate	Tricarboxylic acids	Carboxylic acids	Organic acids
D-Alanine	Other amino acids	Amino acids	Peptides
D-Aspartate	Other amino acids	Amino acids	Peptides
D-Cellobiose	Disaccharides	Disaccharides	Carbohydrates
D-F6P	Sugar 6 phosphates	Sugar phosphates	Carbohydrates
D-Fructose	Ketoses	Monosaccharides	Carbohydrates
D-G1P	Sugar 1 phosphates	Sugar phosphates	Carbohydrates
D-G6P	Sugar 6 phosphates	Sugar phosphates	Carbohydrates
D-Galactose	Aldoses	Monosaccharides	Carbohydrates
D-Galacturonate	Uronic acids	Carboxylic acids	Organic acids
D-GlcNAc	Amino sugars	Monosaccharides	Carbohydrates
D-Galactonate-g-lactone	Esters	Carboxylic acids	Organic acids
D-Gluconate	Sugar acids	Monosaccharides	Carbohydrates
D-Glucosaminate	Aldonic acids	Carboxylic acids	Carbohydrates
D-Glucose	Aldoses	Monosaccharides	Carbohydrates
D-Glucuronate	Dicarboxylic acids	Carboxylic acids	Organic acids
D-Malate	Hydroxycarboxylic acids	Carboxylic acids	Organic acids
D-Mannitol	Sugar alcohols	Monosaccharides	Carbohydrates
D-Mannose	Aldoses	Monosaccharides	Carbohydrates
D-Melibiose	Disaccharides	Disaccharides	Carbohydrates

D-Psicose	Ketoses	Monosaccharides	Carbohydrates
D-Ribose	Aldoses	Monosaccharides	Carbohydrates
D-Saccharate	Dicarboxylic acids	Carboxylic acids	Organic acids
D-Serine	Other amino acids	Amino acids	Peptides
D-Sorbitol	Sugar alcohols	Monosaccharides	Carbohydrates
D-Threonine	Other amino acids	Amino acids	Peptides
D-Trehalose	Disaccharides	Disaccharides	Carbohydrates
D-Xylose	Aldoses	Monosaccharides	Carbohydrates
D,L-G3P	Sugar 3 phosphates	Sugar phosphates	Carbohydrates
D,L-Malate	Hydroxycarboxylic acids	Carboxylic acids	Organic acids
Dulcitol	Sugar alcohols	Monosaccharides	Carbohydrates
Formate	Monocarboxylic acids	Carboxylic acids	Organic acids
Fumarate	Dicarboxylic acids	Carboxylic acids	Organic acids
Glucuronamide	Hexoses	Monosaccharides	Carbohydrates
Glycerol	Sugar 3 phosphates	Sugar phosphates	Carbohydrates
Glycolate	Monocarboxylic acids	Carboxylic acids	Organic acids
Glycyl-L-aspartate	Dipeptides	Amino acids	Peptides
Glycyl-L-glutamate	Dipeptides	Amino acids	Peptides
Glycyl-L-proline	Dipeptides	Amino acids	Peptides
Glyoxylate	2-Oxocarboxylic acids	Carboxylic acids	Organic acids
Inosine	Ribonucleosides	Nucleosides	Nucleic acids
L-Alanine	Common amino acids	Amino acids	Peptides
L-Alanylglycine	Dipeptides	Amino acids	Peptides
L-Arabinose	Aldoses	Monosaccharides	Carbohydrates
L-Asparagine	Common amino acids	Amino acids	Peptides
L-Aspartate	Common amino acids	Amino acids	Peptides
L-Fucose	Deoxy sugars	Monosaccharides	Carbohydrates
L-Galactonate-g-lactone	Esters	Carboxylic acids	Organic acids
L-Glutamate	Common amino acids	Amino acids	Peptides
L-Glutamine	Common amino acids	Amino acids	Peptides
L-Lactate	Hydroxycarboxylic acids	Carboxylic acids	Organic acids
L-Lyxose	Aldoses	Monosaccharides	Carbohydrates
L-Malate	Hydroxycarboxylic acids	Carboxylic acids	Organic acids
L-Proline	Common amino acids	Amino acids	Peptides
L-Rhamnose	Deoxy sugars	Monosaccharides	Carbohydrates
L-Serine	Common amino acids	Amino acids	Peptides
L-Threonine	Common amino acids	Amino acids	Peptides
Lactulose	Disaccharides	Disaccharides	Carbohydrates
M-Acetyl-mannosamine	Hexosamines	Monosaccharides	Carbohydrates

M-Hydroxyphenylacetate	Monocarboxylic acids	Carboxylic acids	Organic acids
M-Inositol	Sugar alcohols	Monosaccharides	Carbohydrates
M-Tartarate	Hydroxycarboxylic acids	Carboxylic acids	Organic acids
Maltose	Disaccharides	Disaccharides	Carbohydrates
Maltotriose	Polysaccharides	Polysaccharides	Carbohydrates
Methylpyruvate	Esters	Carboxylic acids	Organic acids
Methylsuccinate	Dicarboxylic acids	Carboxylic acids	Organic acids
Mucate	Aldaric acids	Carboxylic acids	Organic acids
P-Hydroxyphenylacetate	Monocarboxylic acids	Carboxylic acids	Organic acids
Phenylethylamine	Amines	Amino acids	Peptides
Propionate	Monocarboxylic acids	Carboxylic acids	Organic acids
Pyruvate	2-Oxocarboxylic acids	Carboxylic acids	Organic acids
Succinate	Dicarboxylic acids	Carboxylic acids	Organic acids
Sucrose	Disaccharides	Disaccharides	Carbohydrates
Thymidine	Deoxyribonucleosides	Nucleosides	Nucleic acids
Tricarballylate	Tricarboxylic acids	Carboxylic acids	Organic acids
Tween 20	Polysorbates	Surfactants	Surfactants
Tween 40	Polysorbates	Surfactants	Surfactants
Tween 80	Polysorbates	Surfactants	Surfactants
Tyramine	Amines	Amino acids	Peptides
Uridine	Ribonucleosides	Nucleosides	Nucleic acids

Supplementary Table 3. Carbon source pairings in 32-carbon source experiments (com3, com4, com13). Each individual carbon source combination is numbered and highlighted with alternating background colors, for a total of 63 unique conditions containing varying numbers of carbon sources (nCS). For all communities and individual organisms, experiments were carried out in biological triplicate for each environment.

	nCS = 1, 32 envs.		nCS = 2, 16 envs.		nCS = 4, 8 envs.		nCS = 8, 4 envs.		nCS = 16, 2 envs.		nCS = 32, 1 env.
1	D-Glucose	33	D-Glucose	49	D-Glucose	57	D-Glucose	61	D-Glucose	63	D-Glucose
2	L-Glutamine		D-Sorbitol		D-Sorbitol		D-Sorbitol		D-Sorbitol		D-Sorbitol
3	L-Alanine	34	L-Glutamine		L-Glutamine		L-Glutamine		L-Glutamine		L-Glutamine
4	Pyruvate		Formate		Formate		Formate		Formate		Formate
5	L-Proline	35	L-Alanine	50	L-Alanine		L-Alanine		L-Alanine		L-Alanine
6	D-Trehalose		a-D-Lactose		a-D-Lactose		a-D-Lactose		a-D-Lactose		a-D-Lactose
7	L-Threonine	36	Pyruvate		Pyruvate		Pyruvate		Pyruvate		Pyruvate
8	D-Mannitol		D-G6P		D-G6P		D-G6P		D-G6P		D-G6P
9	D-Fructose	37	L-Proline	51	L-Proline	58	L-Proline		L-Proline		L-Proline
10	D-GlcNAc		D-Galactose		D-Galactose		D-Galactose		D-Galactose		D-Galactose
11	Sucrose	38	D-Trehalose		D-Trehalose		D-Trehalose		D-Trehalose		D-Trehalose
12	Glycerol		D,L-Serine		D,L-Serine		D,L-Serine		D,L-Serine		D,L-Serine
13	D-Gluconate	39	L-Threonine	52	L-Threonine		L-Threonine		L-Threonine		L-Threonine
14	Propionate		D-Xylose		D-Xylose		D-Xylose		D-Xylose		D-Xylose
15	D-Mannose	40	D-Mannitol		D-Mannitol		D-Mannitol		D-Mannitol		D-Mannitol
16	Succinate		L-Arabinose		L-Arabinose		L-Arabinose		L-Arabinose		L-Arabinose
17	Maltose	41	D-Fructose	53	D-Fructose	59	D-Fructose	62	D-Fructose		D-Fructose
18	D,L-Malate		Citrate		Citrate		Citrate		Citrate		Citrate
19	D-Ribose	42	D-GlcNAc		D-GlcNAc		D-GlcNAc		D-GlcNAc		D-GlcNAc
20	L-Lactate		D-Galacturonate		D-Galacturonate		D-Galacturonate		D-Galacturonate		D-Galacturonate
21	D-Cellobiose	43	Sucrose	54	Sucrose		Sucrose		Sucrose		Sucrose
22	Acetate		Acetate		Acetate		Acetate		Acetate		Acetate
23	D-Galacturonate	44	Glycerol		Glycerol		Glycerol		Glycerol		Glycerol
24	Citrate		D-Cellobiose		D-Cellobiose		D-Cellobiose		D-Cellobiose		D-Cellobiose
25	L-Arabinose	45	D-Gluconate	55	D-Gluconate	60	D-Gluconate		D-Gluconate		D-Gluconate
26	D-Xylose		L-Lactate		L-Lactate		L-Lactate		L-Lactate		L-Lactate
27	D,L-Serine	46	Propionate		Propionate		Propionate		Propionate		Propionate
28	D-Galactose		D-Ribose		D-Ribose		D-Ribose		D-Ribose		D-Ribose
29	D-G6P	47	D-Mannose	56	D-Mannose		D-Mannose		D-Mannose		D-Mannose
30	a-D-Lactose		D,L-Malate		D,L-Malate		D,L-Malate		D,L-Malate		D,L-Malate
31	Formate	48	Succinate		Succinate		Succinate		Succinate		Succinate
32	D-Sorbitol		Maltose		Maltose		Maltose		Maltose		Maltose

Supplementary Table 4. Carbon source pairings in 5-carbon source experiments (com3a, com13a). Each background color indicates one environmental composition, for a total of 31 unique conditions containing varying numbers of carbon sources (nCS).

nCS = 1, 5 envs.	nCS = 2, 10 envs.	nCS = 3, 10 envs.	nCS = 4, 5 envs.	nCS = 5, 1 env
D-Glucose	D-Glucose	D-Glucose	D-Glucose	D-Glucose
Pyruvate	Pyruvate	Pyruvate	Pyruvate	Pyruvate
D-GlcNAc	D-Glucose	D-GlcNAc	D-GlcNAc	D-GlcNAc
L-Proline	D-GlcNAc	D-Glucose	L-Proline	L-Proline
L-Threonine	D-Glucose	Pyruvate	D-Glucose	L-Threonine
	L-Proline	L-Proline	Pyruvate	
	D-Glucose	D-Glucose	D-GlcNAc	
	L-Threonine	Pyruvate	L-Threonine	
	Pyruvate	L-Threonine	Pyruvate	
	D-GlcNAc	D-Glucose	D-GlcNAc	
	Pyruvate	D-GlcNAc	L-Proline	
	L-Proline	L-Proline	L-Threonine	
	Pyruvate	D-Glucose	D-Glucose	
	L-Threonine	D-GlcNAc	Pyruvate	
	D-GlcNAc	L-Threonine	L-Proline	
	L-Proline	D-Glucose	L-Threonine	
	D-GlcNAc	L-Proline	D-Glucose	
	L-Threonine	L-Threonine	D-GlcNAc	
	L-Proline	Pyruvate	L-Proline	
	L-Threonine	D-GlcNAc	L-Threonine	
		L-Proline		
		Pyruvate		
		D-GlcNAc		
		L-Threonine		
		D-GlcNAc		
		L-Proline		
		L-Threonine		

Supplementary Table 5. Descriptions of combinatorial carbon source experiments. All experiments were carried out at 30˚C and provided 50 mM C per well (com14 also grown at 25 mM C).

Name	Number of organisms	Organisms	Number of carbon sources	Number of passages	Passaging frequency
com3	3	*B. subtilis*, *M. extorquens*, *S. oneidensis*	32 (Supplementary Table 3)	6	48h
com3a	3	*E. coli*, *M. extorquens*, *S. oneidensis*	5 (Supplementary Table 4)	0 (24h batch)	0
com4	4	com3 + *P. aeruginosa*	32 (Supplementary Table 3)	6	48h
com13	13	com4 + *A. baylyi*, *B. lichenifpormis*, *C. glutamicum*, *E. coli*, *L. lactis*, *P. fluorescens*, *P. putida*, *S. coelicolor*, *V. natriegens*	32 (Supplementary Table 3)	6	48h
com13a	13	com13	5 (Supplementary Table 4)	6	24h
com14	14	com13 + *S. enterica*	4 (Supplementary Figure 3a)	0 (24h batch)	0

Supplementary Table 6. Regression coefficients for individual carbon sources significantly positively or negatively associated with higher Shannon entropy in com13. We generated a simple linear regression model that relates the presence of each carbon source to the Shannon entropy exhibited by the communities. We calculated regression coefficients for each carbon source, allowing us to estimate the contribution of each carbon source to taxonomic balance independent of the number of carbon sources. Here, we estimated D-galactose to be the most highly associated with greater community evenness. Indeed, the environment containing only D-galactose yielded a community with a relatively even composition of *E. coli*, *P. aeruginosa*, and *P. fluorescens*, while the community grown in the two-carbon-source condition with D-galactose also contained three organisms (*A. baylyi*, *P. aeruginosa*, and *P. putida)* (Figure 3a). Despite these relatively balanced communities at lower complexities, the presence of a carbon source significantly associated with lower Shannon entropy like L-arabinose can overpower the effects of carbon sources like D-galactose. This effect is most clearly observed in the 8-carbon source condition containing both these carbon sources, which resulted in the complete dominance of *P. aeruginosa*.

Carbon source	Coefficient	P-value
L-Glutamine	0.619	2.78E-02
D,L-Malate	0.858	2.40E-03
D-Ribose	0.795	4.90E-03
D-Cellobiose	1.049	2.00E-04
Citrate	1.049	2.00E-04
D-Xylose	0.673	1.69E-02
D-Galactose	0.752	7.70E-03
D-Glucose	-0.826	3.50E-03
D-Fructose	-0.951	8.00E-04
Glycerol	-1.285	0.00E+00
D-Gluconate	-0.872	2.10E-03
Propionate	-1.205	0.00E+00
L-Arabinose	-1.105	1.00E-04

Supplementary Table 7. Descriptions and quantities for consumer resource model state variables and parameters. The initial resource concentration is based on the 50 mM C of glucose used in our experiments.

Variable/parameter	Description	Units	Quantity
N_i	Abundance of organism i	CFU/mL	variable
R_α	Abundance of resource α	g/mL	variable
$C_{i\alpha}$	Uptake rate per unit concentration of resource α by organism i	mL/hr	variable
$D_{\alpha\beta i}$	Proportion of resource α converted to resource β by organism i	unitless	variable
l_α	Leakage fraction for resource α	unitless	0.25 [7]
g_i	Conversion factor from energy uptake to growth rate for organism i	1/energy	1 [7]
w_α	Energy content of resource α	energy/g	2.5×10^9
$k_{i,a}$	Half velocity constant for resource uptake	g/mL	1×10^4
m_i	Minimal energy uptake for maintenance of each species i	energy/hr	0.05
κ_α	External supply of resource α	g/mL/hr	$1.5 g/mL$

Supplementary Table 8. Minimal medium composition for flux-balance modeling.

Metabolite	BIGG metabolite name[25]
Calcium	ca2[e]
Chloride	cl[e]
Cobalt	cobalt2[e]
$Copper^{2+}$	cu2[e]
$Iron^{2+}$	fe2[e]
$Iron^{3+}$	fe3[e]
Hydrogen	h2[e]
Water	h2o[e]
Potassium	k[e]
Magnesium	mg2[e]
Manganese	mn2[e]
Molybdate	mobd[e]
Sodium	na1[e]
Ammonium	nh4[e]
$Nickel^{2+}$	ni2[e]
Nitrate	no3[e]
Oxygen	o2[e]
Phosphate	pi[e]
Sulfate	so4[e]
$Zinc^{2+}$	zn2[e]

Supplementary Table 9. List of unique organic molecules predicted to be secreted across all flux-balance simulations.

**Metabolite**	**BIGG metabolite name**[25]
Acetate	ac[e]
D-alanine	ala-D[e]
Allantoin	alltn[e]
Citrate	cit[e]
Formate	for[e]
Formamide	frmd[e]
Fumarate	fum[e]
Glycolaldehyde	gcald[e]
D-gluconate	glcn-D[e]
Glycolate	glyclt[e]
L-lactate	lac-L[e]
D-malate	mal-D[e]
R-pantothenate	pnto-R[e]
Riboflavin	ribflv[e]
Spermidine	spmd[e]
Succinate	succ[e]
Trehalose	tre[e]
Urea	urea[e]

References

1. Pacheco, A. R., Moel, M. & Segrè, D. Costless metabolic secretions as drivers of interspecies interactions in microbial ecosystems. *Nature Communications* **10**, 103 (2019).
2. Replansky, T. & Bell, G. The relationship between environmental complexity, species diversity and productivity in a natural reconstructed yeast community. *Oikos* **118**, 233–239 (2009).
3. Fonte, E. S. *et al.* The Combination of Different Carbon Sources Enhances Bacterial Growth Efficiency in Aquatic Ecosystems. *Microbial Ecology* **66**, 871–878 (2013).
4. Wang, X., Xia, K., Yang, X. & Tang, C. Growth strategy of microbes on mixed carbon sources. *Nature Communications* **10**, 1–7 (2019).
5. Goldford, J. E. *et al.* Emergent simplicity in microbial community assembly. *Science* **361**, 469–474 (2018).
6. San Roman, M. & Wagner, A. An enormous potential for niche construction through bacterial cross-feeding in a homogeneous environment. *PLoS Computational Biology* **14**, e1006340 (2018).
7. Marsland, R., Cui, W. & Mehta, P. A minimal model for microbial biodiversity can reproduce experimentally observed ecological patterns. *Scientific Reports* **10**, 1–17 (2020).
8. Henry, C. S., Zinner, J. F., Cohoon, M. P. & Stevens, R. L. iBsu1103: a new genome-scale metabolic model of Bacillus subtilis based on SEED annotations. *Genome Biology* **10**, R69 (2009).
9. Peyraud, R. *et al.* Genome-scale reconstruction and system level investigation of the metabolic network of Methylobacterium extorquens AM1. *BMC Systems Biology* **5**, (2011).
10. Oberhardt, M. A., Puchałka, J., Fryer, K. E., Martins dos Santos, V. A. P. & Papin, J. A. Genome-scale metabolic network analysis of the opportunistic pathogen Pseudomonas aeruginosa PAO1. *Journal of Bacteriology* **190**, 2790–803 (2008).
11. Pinchuk, G. E. *et al.* Constraint-Based Model of Shewanella oneidensis MR-1 Metabolism: A Tool for Data Analysis and Hypothesis Generation. *PLoS Computational Biology* **6**, e1000822 (2010).
12. Bordbar, A., Monk, J. M., King, Z. A. & Palsson, B. O. Constraint-based models predict metabolic and associated cellular functions. *Nature Reviews Genetics* **15**, 107–120 (2014).
13. Orth, J. D., Thiele, I. & Palsson, B. Ø. O. What is flux balance analysis? *Nature Biotechnology* **28**, 245–248 (2010).
14. Heirendt, L. *et al.* Creation and analysis of biochemical constraint-based models using the COBRA Toolbox v.3.0. *Nature Protocols* **14**, 639–702 (2019).
15. Holzhütter, H.-G. The principle of flux minimization and its application to estimate stationary fluxes in metabolic networks. *European Journal of Biochemistry* **271**, 2905–2922 (2004).
16. Eguíluz, V. M. *et al.* Scaling of species distribution explains the vast potential marine prokaryote diversity. *Scientific Reports* **9**, 18710 (2019).
17. Locey, K. J. & Lennon, J. T. Scaling laws predict global microbial diversity. *Proceedings of the National Academy of Sciences of the United States of America* **113**, 5970–5 (2016).
18. Hoffmann, K. H. *et al.* Power law rank-abundance models for marine phage communities. *FEMS Microbiology Letters* **273**, 224–8 (2007).
19. Durot, M. *et al.* Iterative reconstruction of a global metabolic model of Acinetobacter baylyi ADP1 using high-throughput growth phenotype and gene essentiality data. *BMC Systems Biology* **2**, 85 (2008).
20. Orth, J. D. *et al.* A comprehensive genome-scale reconstruction of Escherichia coli metabolism—2011. *Molecular Systems Biology* **7**, 535 (2011).
21. Flahaut, N. A. L. *et al.* Genome-scale metabolic model for Lactococcus lactis MG1363 and its application to the analysis of flavor formation. *Applied Microbiology and Biotechnology* **97**, 8729–8739 (2013).

22. Nogales, J., Palsson, B. Ø. & Thiele, I. A genome-scale metabolic reconstruction of Pseudomonas putida KT2440: iJN746 as a cell factory. *BMC Systems Biology* **2**, 79 (2008).
23. Kim, M. *et al.* Reconstruction of a high-quality metabolic model enables the identification of gene overexpression targets for enhanced antibiotic production in Streptomyces coelicolor A3(2). *Biotechnology Journal* **9**, 1185–1194 (2014).
24. Thiele, I. *et al.* A community effort towards a knowledge-base and mathematical model of the human pathogen Salmonella Typhimurium LT2. *BMC Systems Biology* **5**, 8 (2011).
25. King, Z. A. *et al.* BiGG Models: A platform for integrating, standardizing and sharing genome-scale models. *Nucleic Acids Research* **44**, D515–D522 (2016).
