# Supplementary material for: Non-additive microbial community responses to environmental complexity
Source: Nat Commun. 2021 Apr 22;12:2365. doi: 10.1038/s41467-021-22426-3 (PMC8062479; doi:10.1038/s41467-021-22426-3)
Supplement: Supplementary file 3 — Description of Additional Supplementary Files [file 41467_2021_22426_MOESM3_ESM.pdf]

Description of Additional Supplementary Files

File Name: Supplementary Data 1
Description: Categorized community taxonomic outcomes on combinations of carbon sources.
